# MDA5-dependent responses contribute to autoimmune diabetes progression and hindrance

**DOI:** 10.1172/jci.insight.157929

**Published:** 2023-01-24

**Authors:** Samuel I. Blum, Jared P. Taylor, Jessie M. Barra, Ashley R. Burg, Qiao Shang, Shihong Qiu, Oren Shechter, Aleah R. Hayes, Todd J. Green, Aron M. Geurts, Yi-Guang Chen, Hubert M. Tse

**Affiliations:** 1 Department of Microbiology, Comprehensive Diabetes Center, The University of Alabama at Birmingham, Birmingham, Alabama, USA.; 2 Department of Physiology and; 3 Department of Pediatrics, Medical College of Wisconsin, Milwaukee, Wisconsin, USA.

**Keywords:** Autoimmunity, Virology, Diabetes, Innate immunity, T cells

## Abstract

Type 1 diabetes (T1D) is an autoimmune disease resulting in pancreatic *β* cell destruction. Coxsackievirus B3 (CVB3) infection and melanoma differentiation-associated protein 5–dependent (MDA5-dependent) antiviral responses are linked with T1D development. Mutations within *IFIH1*, coding for MDA5, are correlated with T1D susceptibility, but how these mutations contribute to T1D remains unclear. Utilizing nonobese diabetic (NOD) mice lacking *Ifih1* expression (*KO*) or containing an in-frame deletion within the ATPase site of the helicase 1 domain of MDA5 (Δ*Hel1*), we tested the hypothesis that partial or complete loss-of-function mutations in MDA5 would delay T1D by impairing proinflammatory pancreatic macrophage and T cell responses. Spontaneous T1D developed in female NOD and *KO* mice similarly, but was significantly delayed in Δ*Hel1* mice, which may be partly due to a concomitant increase in myeloid-derived suppressor cells. Interestingly, *KO* male mice had increased spontaneous T1D compared with NOD mice. Whereas NOD and *KO* mice developed CVB3-accelerated T1D, Δ*Hel1* mice were protected partly due to decreased type I IFNs, pancreatic infiltrating TNF^+^ macrophages, IFN-*γ*^+^CD4^+^ T cells, and perforin^+^CD8^+^ T cells. Furthermore, Δ*Hel1* MDA5 protein had reduced ATP hydrolysis compared with wild-type MDA5. Our results suggest that dampened MDA5 function delays T1D, yet loss of MDA5 promotes T1D.

## Introduction

Type 1 diabetes (T1D) is a T cell–mediated autoimmune disease resulting in pancreatic β cell destruction (1). A synergistic effect of genetics, the environment, and the immune system is proposed to induce T1D (2–5). Monozygotic twins have a ≈30%–50% concordance rate for T1D, which suggests that the environment plays a major role in T1D development (6, 7). One environmental factor associated with T1D is coxsackievirus B (CVB) infection (8, 9). CVB viral RNA and/or virus particles have been detected in the blood, stool, and pancreatic islets of patients with recent-onset T1D (9–11). In the nonobese diabetic (NOD) mouse model, CVB infections accelerate T1D by inducing inflammatory pancreatic antiviral responses resulting in β cell destruction (12, 13).

The innate viral sensor melanoma differentiation-associated protein 5 (MDA5), encoded by the *IFIH1* gene, detects dsRNA viral replication intermediates and initiates antiviral signaling (14, 15). One of the key responses of MDA5 after binding its ligand is the synthesis of type I IFNs, such as IFN-α and IFN-β, to promote viral clearance and activation of macrophages, dendritic cells, and T cells (16–20). Although type I IFNs are crucial to antiviral responses, they have also been linked to early T1D development (21, 22). In transgenic CD1 mice, where β cells constitutively express IFN-α, T1D onset occurs for 60% of the mice by 10 weeks of age (23). In contrast, loss of IFN-α and -β receptor subunit 1 (IFNAR1) expression in NOD female mice results in a significant delay in T1D development (24). In patients with T1D, a type I IFN gene signature is detected in the blood prior to autoantibody development (21, 22), and GWAS have found genes associated with T1D that are involved in type I IFN synthesis and signaling, such as *IFIH1* (25, 26).

Multiple single nucleotide polymorphisms (SNPs) within *IFIH1* are associated with human T1D development. The A946T SNP (rs1990760), which results in an alanine-to-threonine change at amino acid 946, is associated with T1D risk and leads to increased IFN-α/β and IFN-stimulated gene production by human peripheral blood mononuclear cells (27, 28). Mice carrying the A946T SNP are protected from a lethal viral challenge but at the cost of increased susceptibility to autoimmunity (27). Conversely, CVB3 infection of human islets homozygous for the A946T SNP results in decreased type III IFN production and improved viral clearance (29). These seemingly contradictory findings show that further studies are required to fully understand how mutations in *IFIH1* result in an increased risk for developing T1D.

In contrast, some *IFIH1* SNPs are associated with protection from T1D, such as I923V (rs35667974), which results in an isoleucine-to-valine change at amino acid (AA) 923, and E627x (rs35744605), which results in a nonsense mutation and an early stop codon at AA 627 (30). The I923V SNP results in reduced type I IFN synthesis and ATP hydrolysis and increased dsRNA dissociation (31). The E627x SNP causes reduced MDA5 expression and reduced type I IFN synthesis (32). Lincez et al. previously demonstrated that reduced MDA5 expression in NOD.MDA5^+/–^ mice delays spontaneous and CVB type 4–accelerated (CVB4-accelerated) T1D partly due to enhanced regulatory T cells (Tregs) and reduced effector CD4^+^ T cells in the pancreatic lymph nodes (PLNs), which correlated with reduced pancreatic *Ifna* mRNA (13). However, the role of MDA5 on macrophage and T cell responses within the pancreata during spontaneous and CVB-accelerated T1D remains unclear.

To further investigate MDA5-dependent antiviral responses in T1D, we used zinc finger nuclease genomic editing to introduce mutations in the helicase 1 domain of MDA5 in NOD mice to recapitulate *IFIH1* SNPs that cause reduced MDA5 expression and are associated with a delay in T1D progression (30). We generated NOD mice with an in-frame 5 AA deletion in the helicase 1 domain of MDA5 (*ΔHel1*) and an out-of-frame deletion resulting in a premature stop codon in MDA5 (*KO*). Interestingly, the *KO* mutation does not lead to any detectable truncated MDA5 protein. The helicase 1 domain senses dsRNA, contains ATPase activity, and interacts with the caspase activation recruitment domain (CARD) to promote antiviral responses (30). We used these 2 mouse models to explore the effects of mutations in MDA5 in spontaneous and CVB3-accelerated T1D. We hypothesized that partial or complete loss-of-function mutations in MDA5 would delay T1D onset by impairing proinflammatory pancreatic macrophage and T cell responses.

Our results show that mutations in MDA5 can influence both spontaneous and CVB3-accelerated T1D. Interestingly, *KO* mice had no protection from spontaneous or CVB3-accelerated T1D. Male *KO* mice developed spontaneous T1D at a faster rate compared with NOD male mice. Conversely, *ΔHel1* mice had a delay in spontaneous and CVB3-accelerated T1D, partly due to reductions in proinflammatory pancreatic macrophages and T cells. Furthermore, purified *ΔHel1* MDA5 protein had reduced ATPase activity compared with wild-type (WT) MDA5 protein. Our data indicate that protection from T1D may be partially intrinsic to reduced MDA5 function and type I IFN synthesis.

## Results

### Ifih1^ΔHel1^ and Ifih1^KO^ mutations affect T1D disease progression in NOD mice.

**Figure 1:**
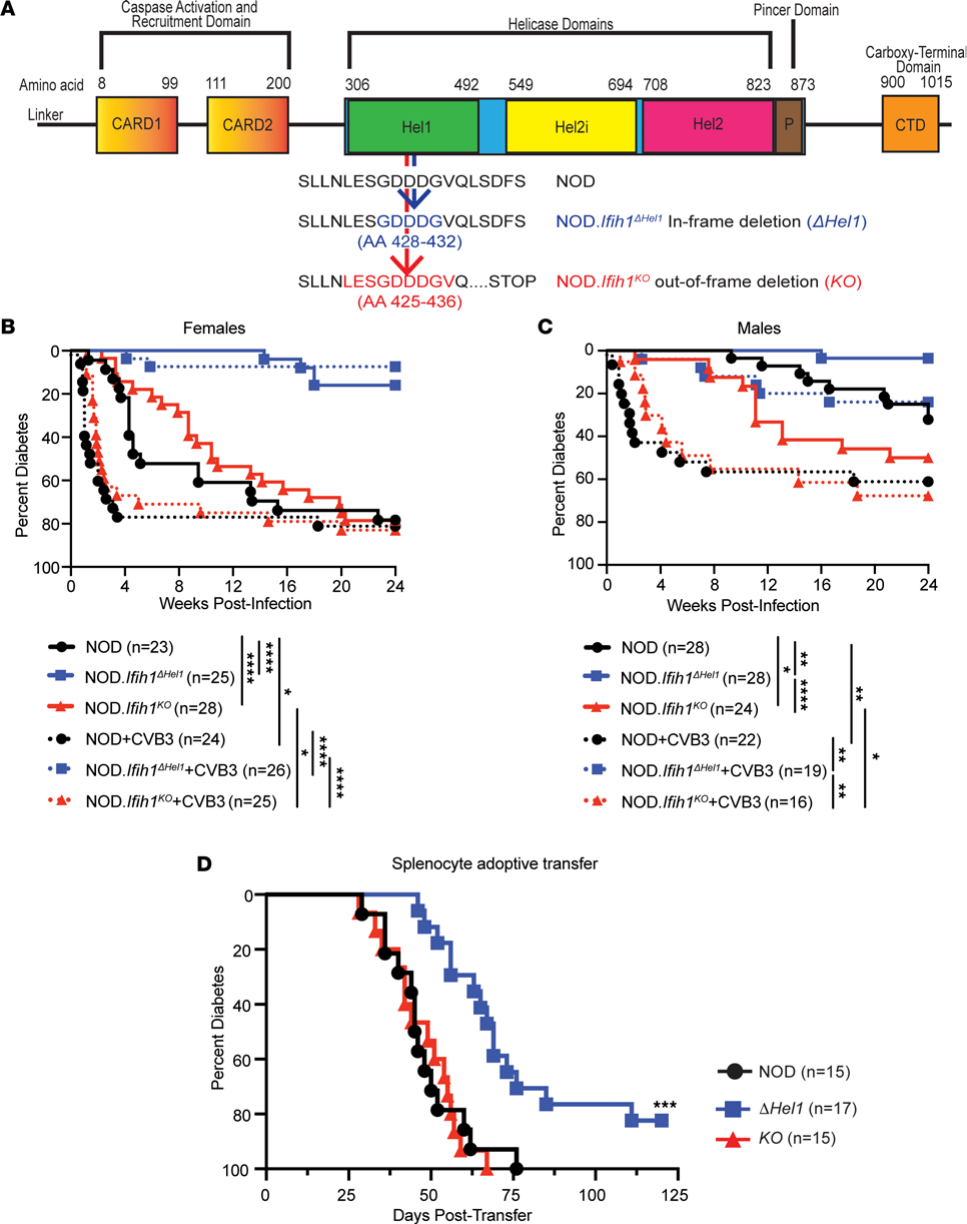
*Ifih1^ΔHel1^* and *Ifih1^KO^* mutations affect T1D disease progression in NOD mice. Diagram of MDA5 containing CARD1, CARD2, helicase1 (Hel1), helicase2i (Hel2i), helicase2 (Hel2), and carboxy terminal domain (CTD). *Ifih1^ΔHel1^* (*ΔHel1*) and *Ifih1^KO^* (*KO*) mutations are shown within the helicase 1 domain of MDA5 (**A**). Kaplan-Meier survival curve of diabetes incidence of uninfected and CVB-infected NOD, *ΔHel1*, and *KO* female mice (**B**) and male mice (**C**). Kaplan-Meier survival curve of NOD.*Rag* mice that received an i.v. adoptive transfer of 10^7^ nondiabetic NOD splenocytes (**D**). Analyzed by log-rank (Mantel-Cox) test (**B**–**D**). *P < 0.05, **P < 0.01, ***P < 0.001, ****P < 0.0001. (**B**) *n* = 23–28, (**C**) *n* = 16–28, and (**D**) *n* = 15–17.

To identify how the loss of MDA5 expression affected T1D development, NOD mice with mutations in *Ifih1* were generated by genomic editing with zinc finger nucleases targeting the helicase 1 domain of MDA5. We generated NOD.*Ifih1*^ΔHel1^ (*ΔHel1*) mice with an in-frame deletion at AA 428–432 and NOD.*Ifih1^KO^* (*KO*) mice with an out-of-frame deletion at AA 425–436 resulting in the generation of a premature stop codon (Figure 1A). The effects of *Ifih1*^ΔHel1^ and *Ifih1^KO^* mutations on spontaneous autoimmune and virus-accelerated diabetes were assessed in male and female NOD, *ΔHel1*, and *KO* mice. Uninfected female NOD and *KO* mice developed T1D similarly, whereas T1D development in *ΔHel1* mice was significantly (*P* < 0.0001) delayed (Figure 1B). Uninfected male *ΔHel1* mice also exhibited significant delays in T1D compared with NOD (*P* < 0.01) and *KO* (*P* < 0.0001) mice (Figure 1C). Interestingly, uninfected male *KO* mice had significant (*P* < 0.05) acceleration of T1D compared with NOD mice (Figure 1C), highlighting the potentially novel role of MDA5 to promote or delay T1D.

To evaluate the effects of the *Ifih1* mutations on virus-accelerated T1D, we infected 12-week-old female and male NOD, *ΔHel1*, and *KO* mice with 100 PFU of CVB3/Woodruff and monitored for T1D. CVB3 infected female NOD and *KO* mice displayed a significant (*P* < 0.05) acceleration of T1D and became diabetic as early as 1 week postinfection (Figure 1B). However, female *ΔHel1* mice were significantly (*P* < 0.0001) delayed from CVB3-accelerated T1D compared with infected NOD and *KO* mice (Figure 1B). CVB3-infected male NOD (*P* < 0.01) and *KO* (*P* < 0.05) mice also displayed a significant acceleration of T1D, whereas CVB3-infected male *ΔHel1* mice were significantly (*P* < 0.01) delayed compared with infected NOD and *KO* mice (Figure 1C).

To assess if the delay in spontaneous T1D observed in *ΔHel1* mice was due to diminished immune responses, we performed an adoptive transfer with splenocytes from euglycemic female NOD, *ΔHel1*, and *KO* mice into NOD.*Rag* recipients. NOD and *KO* splenocytes induced T1D similarly in recipient mice, but the kinetics of disease transfer with *ΔHel1* splenocytes was significantly (*P* < 0.001) delayed (Figure 1D). The *Ifih1*^ΔHel1^ mutation reduced diabetogenicity of immune cells, but the *Ifih1^KO^* mutation did not abrogate autoimmune responses.

### Ifih1 mutations dampen islet infiltration without hindering insulin secretion.

To determine if the *Ifih1*^ΔHel1^ and *Ifih1^KO^* mutations affected glucose homeostasis and β cell function, intraperitoneal glucose tolerance test (IPGTT) and glucose-stimulated insulin secretion (GSIS) assays were performed on NOD, *ΔHel1*, and *KO* mice. No differences in glucose clearance were observed by IPGTT, as all mice returned to euglycemia by 120 minutes postinjection (Supplemental Figure 1, A–D; supplemental material available online with this article; https://doi.org/10.1172/jci.insight.157929DS1). To validate these results, we performed a GSIS assay on islets, and no differences were observed in insulin secretion (Supplemental Figure 1, E and F). Furthermore, no changes were observed in the body weight of male and female NOD and *ΔHel1* mice. Female *KO* mice had significantly (*P* < 0.001) reduced body weight compared with *ΔHel1* mice but not NOD mice (Supplemental Figure 1G). The decreased body weight in female *KO* mice did not compromise their ability to thrive, as negative effects on health were not observed. Finally, no differences in body weight were observed in male mice (Supplemental Figure 1H).

**Figure 2:**
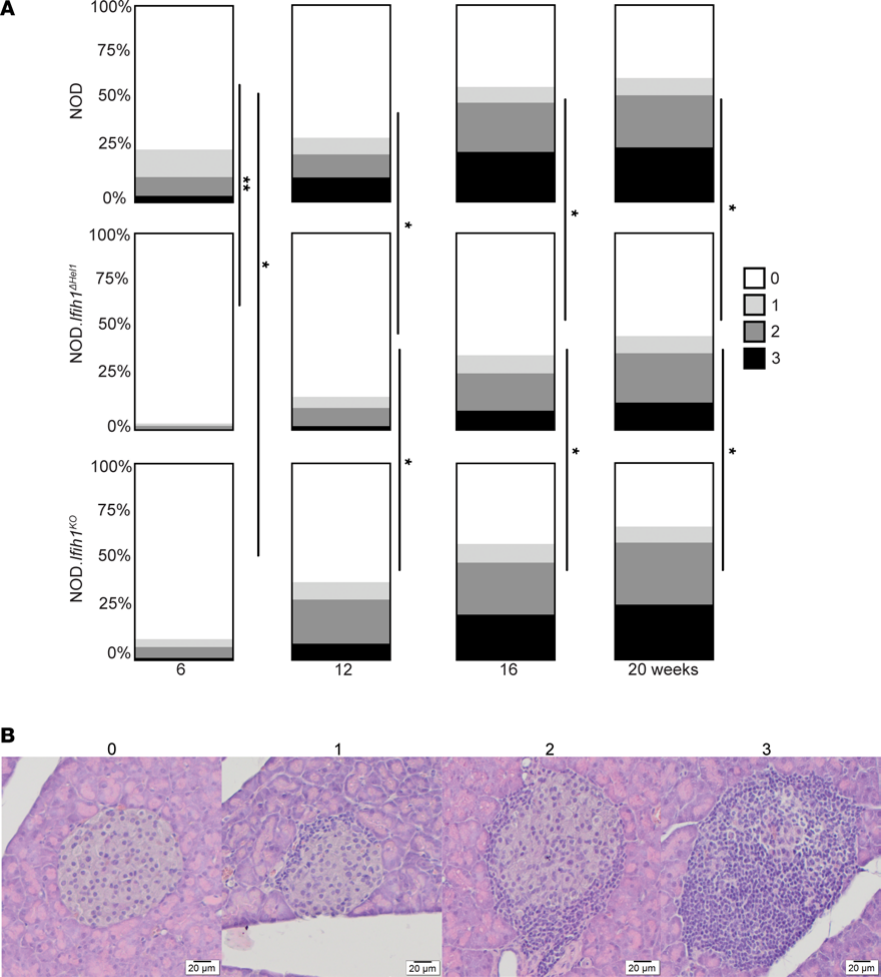
*ΔHel1* mice have reduced islet infiltration. Insulitis scoring of islets from NOD, *ΔHel1*, and *KO* mice at 6, 12, 16, and 20 weeks of age (**A**). Representative H&E images of islets used for insulitis scoring (**B**). Analyzed by 2-way ANOVA with Tukey’s multiple comparisons (**A**). *P < 0.05, **P < 0.01. (**A** and **B**) *n* = 5 with 80–140 islets per sample.

We next performed insulitis scoring on NOD, *ΔHel1*, and *KO* mice to assess differences in pancreatic islet infiltration. *ΔHel1* and *KO* islets had a significant (*P* < 0.05 and *P* < 0.01, respectively) reduction in immune cell infiltration compared with NOD islets at 6 weeks of age. At 12, 16, and 20 weeks of age, insulitis scores from *ΔHel1* mice were significantly (*P* < 0.05) reduced compared with NOD and *KO* mice (Figure 2, A and B). The *Ifih1*^ΔHel1^ mutation delayed T1D development partly due to reduced immune cell infiltration of islets without compromising β cell function.

### Mutations in Ifih1 lead to reduced pancreatic proinflammatory macrophage and T cell populations.

Since CVB has a tropism for the pancreas and can induce macrophage and T cell infiltration, leading to the destruction of infected pancreatic exocrine and endocrine cells (33–35), we investigated if the *Ifih1*^ΔHel1^ and *Ifih1^KO^* mutations affected pancreatic macrophage and T cell populations. We analyzed pancreatic macrophages from both uninfected and CVB3-infected NOD, *ΔHel1*, and *KO* female mice by flow cytometry at 7 days postinfection. Pancreatic macrophage (F4/80^+^CD11b^+^) frequency and cell counts were unaltered between uninfected NOD, *ΔHel1*, and *KO* mice (Supplemental Figure 2, A and B). However, there was a significant increase in F4/80^+^CD11b^+^ macrophage cell counts following CVB3 infection compared with uninfected controls (Supplemental Figure 2B), while frequency remained unaltered (Supplemental Figure 2A).

**Figure 3:**
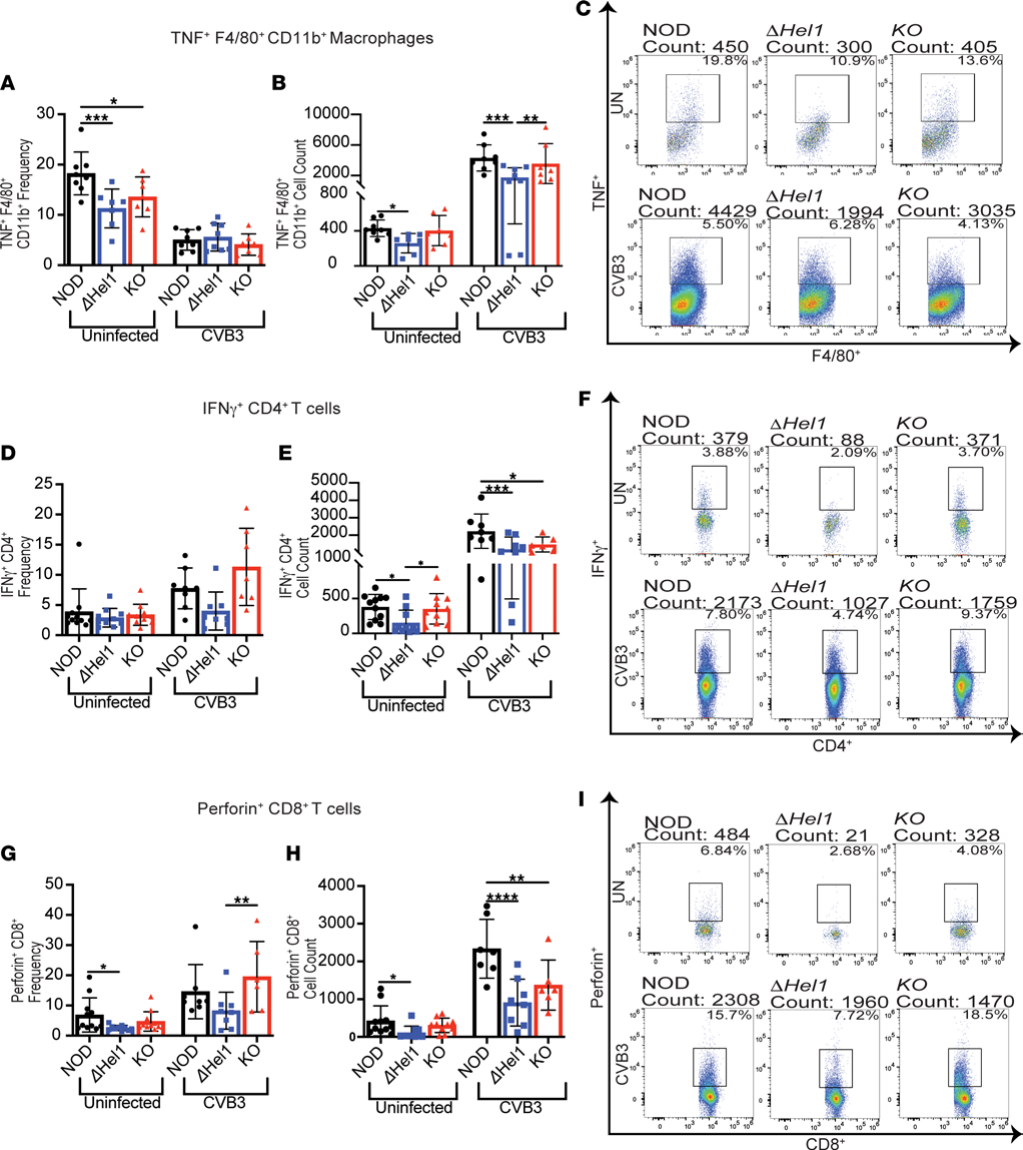
Mutations in *Ifih1* lead to reduced pancreatic proinflammatory macrophage and T cell populations. Flow cytometry analysis of pancreas-infiltrating macrophages, CD4^+^ T cell, and CD8^+^ T cell intracellular cytokine synthesis from uninfected and CVB3-infected NOD, *ΔHel1*, and *KO* female mice at day 7 postinfection. Flow cytometric analysis of TNF^+^F4/80^+^CD11b^+^ macrophage frequency (**A**), cell counts (**B**), and representative flow plots (**C**). Flow cytometric analysis for IFN-γ^+^CD4^+^ T cell frequency (**D**), cell counts (**E**), and representative flow plot (**F**). Flow cytometric analysis for perforin^+^CD8^+^ T cell frequency (**G**), cell counts (**H**), and representative flow plot (**I**). Analyzed by 1-way ANOVA with Tukey’s multiple comparisons (**A**, **B**, **E**, **G**, and **H**). *P < 0.05, **P < 0.01, ***P < 0.001, and ****P < 0.0001. *n* = 6–8.

The activation status of macrophages was determined by MHC-II, CD80, and TNF expression. We observed no significant differences in frequency and cell count of activated CD80^+^F4/80^+^CD11b^+^ macrophages and MHC-II^+^F4/80^+^CD11b^+^ macrophages between all groups of uninfected and CVB3-infected mice (data not shown). However, there was a significant reduction in TNF^+^F4/80^+^CD11b^+^ macrophage frequency in uninfected *ΔHel1* (≈1.6-fold, *P* < 0.001) and *KO* (≈1.3-fold, *P* < 0.05) mice compared with uninfected NOD mice (Figure 3, A and C). Uninfected *ΔHel1* mice also had a ≈1.6-fold (*P* < 0.05) decrease in cell count compared with uninfected NOD, but no differences were observed between uninfected NOD and *KO* mice (Figure 3, B and C). At day 7 postinfection, pancreatic TNF^+^F4/80^+^CD11b^+^ macrophage cell counts from CVB3-infected *ΔHel1* mice were significantly reduced when compared with NOD (≈2.5-fold, *P* < 0.001) and *KO* (≈2.0-fold, *P* < 0.01) mice (Figure 3, B and C). However, no differences in frequency were observed following CVB3 infection; this may be due to the large influx of macrophages into NOD and *KO* pancreata compared with *ΔHel1* mice (Figure 3, A and C). Therefore, loss of MDA5 in *KO* mice did not alter inflammatory macrophages, while both uninfected and CVB3-infected *ΔHel1* mice had a reduction in proinflammatory macrophages within the pancreata, which may partly explain the delay in both spontaneous and CVB3-accelerated T1D.

Because the *Ifih1*^ΔHel1^ mutation decreased proinflammatory macrophage responses, we next examined the effect on pancreatic T cell effector responses from CVB3-infected NOD, *ΔHel1*, and *KO* mice at 7 days postinfection. Following CVB3 infection, *KO* mice had significantly fewer (≈1.8-fold, *P* < 0.05) CD4^+^ T cells within the pancreata compared with infected NOD and *ΔHel1* mice (Supplemental Figure 2D) and significantly fewer (≈1.6-fold, *P* < 0.05) CD8^+^ T cells compared with infected NOD mice (Supplemental Figure 2F). However, no differences in the frequency or cell counts of total pancreas-infiltrating CD4^+^ or CD8^+^ T cells were observed between uninfected NOD, *ΔHel1*, or *KO* mice (Supplemental Figure 2, C–F). Furthermore, we observed no significant differences between activated CD69^+^CD4^+^ or CD69^+^CD8^+^ T cells between uninfected or CVB3-infected NOD, *ΔHel1*, and *KO* mice (data not shown).

Conversely, the effector response of pancreatic CD4^+^ and CD8^+^ T cells was different in mice containing *Ifih1*^ΔHel1^ and *Ifih1^KO^* mutations. Uninfected NOD and *KO* mice had similar frequencies and cell counts of IFN-γ^+^CD4^+^ T cells (Figure 3, D–F) and following phorbol 12-myristate 13-acetate and ionomycin (PMA/I) stimulation (Supplemental Figure 3A). Conversely, *ΔHel1* mice had a significant reduction in pancreatic IFN-γ^+^CD4^+^ T cell counts compared with NOD (≈2.4-fold, *P* < 0.05) and *KO* (≈2.3-fold, *P* < 0.05) mice (Figure 3, E and F). Similar decreases were also observed with IFN-γ^+^CD4^+^ T cells from *ΔHel1* mice compared with NOD (≈2.3-fold, *P* < 0.05) and *KO* (≈2.1-fold, *P* < 0.05) mice following PMA/I stimulation (Supplemental Figure 3A). Even though there was no statistical difference in the frequency of IFN-γ^+^CD4^+^ T cells from *ΔHel1* mice, the mean frequency of IFN-γ^+^CD4^+^ T cells from *ΔHel1* mice was reduced compared with NOD and *KO* (Figure 3, D and F, and Supplemental Figure 3A). At day 7 postinfection with CVB3, there were no significant differences in the effector response of IFN-γ^+^CD4^+^ T cell frequencies between uninfected or CVB3-infected NOD, *ΔHel1*, and *KO* mice, but the mean frequency was reduced in *ΔHel1* mice compared with CVB3-infected NOD and *KO* (Figure 3, D and F). With respect to cell numbers, CVB3-infected *ΔHel1* (≈1.9-fold, *P* < 0.001) and *KO* (≈1.5-fold, *P* < 0.05) mice had significantly fewer pancreatic IFN-γ^+^CD4^+^ T cells compared with NOD mice (Figure 3, E and F).

Uninfected *ΔHel1* mice also had a significant reduction in pancreatic perforin^+^CD8^+^ T cell frequency (≈2.7-fold, *P* < 0.05) and cell count (≈3.9-fold, *P* < 0.05) compared with NOD, but no difference was observed compared to *KO* mice (Figure 3, G–I). Following CVB3 infection, we observed that *KO* mice had a significant increase (≈1.3-fold, *P* < 0.01) in perforin^+^CD8^+^ T cell frequency compared with CVB3-infected *ΔHel1* mice, but no difference between CVB3-infected NOD and *ΔHel1* mice was observed (Figure 3, G and I). CVB3-infected *ΔHel1* (≈2.6-fold, *P* < 0.0001) and *KO* (≈1.7-fold, *P* < 0.01) mice had significantly fewer pancreatic perforin^+^CD8^+^ T cells compared with infected NOD mice (Figure 3, H and I). The discrepancy between frequency and cell count of IFN-γ^+^CD4^+^ and perforin^+^CD8^+^ T cells from CVB3-infected *KO* mice was due to significantly fewer total CD4^+^ and CD8^+^ T cells within the pancreata of *KO* mice compared with NOD mice.

CVB3-infected *ΔHel1* and *KO* mice had similar reductions in IFN-γ^+^CD4^+^ and perforin^+^CD8^+^ T cell counts, indicating that MDA5-dependent antiviral responses are necessary for efficient T cell effector responses. However, uninfected *ΔHel1* mice had fewer IFN-γ^+^CD4^+^ and perforin^+^CD8^+^ T cells, which may explain the delay in spontaneous T1D development. Furthermore, Tregs play a critical role in peripheral tolerance and delaying T1D, but the frequency and cell counts of pancreatic CD25^+^FoxP3^+^CD4^+^ T cells were unaltered in NOD, *ΔHel1*, and *KO* mice (Supplemental Figure 3B). However, CD25^+^FoxP3^+^CD4^+^ T cell frequency was significantly (≈1.2-fold, *P* < 0.05) reduced in *KO* PLNs compared with *ΔHel1* mice during T1D development and was unaltered compared to NOD (Supplemental Figure 3C). Reduced Tregs within the PLNs of *KO* mice may partly explain the inability of these mice to delay spontaneous T1D (Figure 1, B and C).

### Ifih1^ΔHel1^ mutation enhances myeloid-derived suppressor cell populations.

One subset of innate immune cells that can regulate proinflammatory macrophages and T cells are myeloid-derived suppressor cells (MDSCs) (36, 37). MDSCs are either neutrophil like (PMN-MDSCs) or monocyte like (M-MDSCs) and have potent immune suppressive function via arginase-1, nitric oxide synthase, reactive oxygen species, IL-10, TGF-β, IL-1β, and programmed cell death ligand 1 (38–40). These suppressor cells have been suggested to play a major role in preventing T1D. NOD mice adoptively transferred with MDSCs are protected from T1D development (41), and patients with T1D are reported to have reduced MDSC suppressive activity compared with healthy controls (42).

Studies in pancreatic cancer have suggested a link between MDA5, type I IFN signaling, and MDSC function, but type I IFNs can have divergent effects on MDSC function. Too much type I IFN signaling can result in dampened MDSC suppressor activity, whereas a complete loss of type I IFNs can result in impaired MDSC development (43–45). Previous studies have shown that poly(I:C) stimulation of MDA5 in MDSCs induces type I IFN synthesis, which dampens their suppressive capacity (45, 46). However, a complete loss of IFNAR1 on MDSCs prevents their development and suppressive activity (43–45). These findings provide evidence that an optimal amount of type I IFN activity is necessary for MDSC development and function. Interestingly, reduced surface expression of IFNAR1 on MDSCs may increase their suppressive activity by reducing type I IFN signaling (43), suggesting that fine-tuning of type I IFN signaling may affect MDSC function. Given the importance of MDA5 and type I IFNs on MDSC suppressive function and development, we hypothesized that MDSC populations may be altered within *ΔHel1* and *KO* mice.

**Figure 4:**
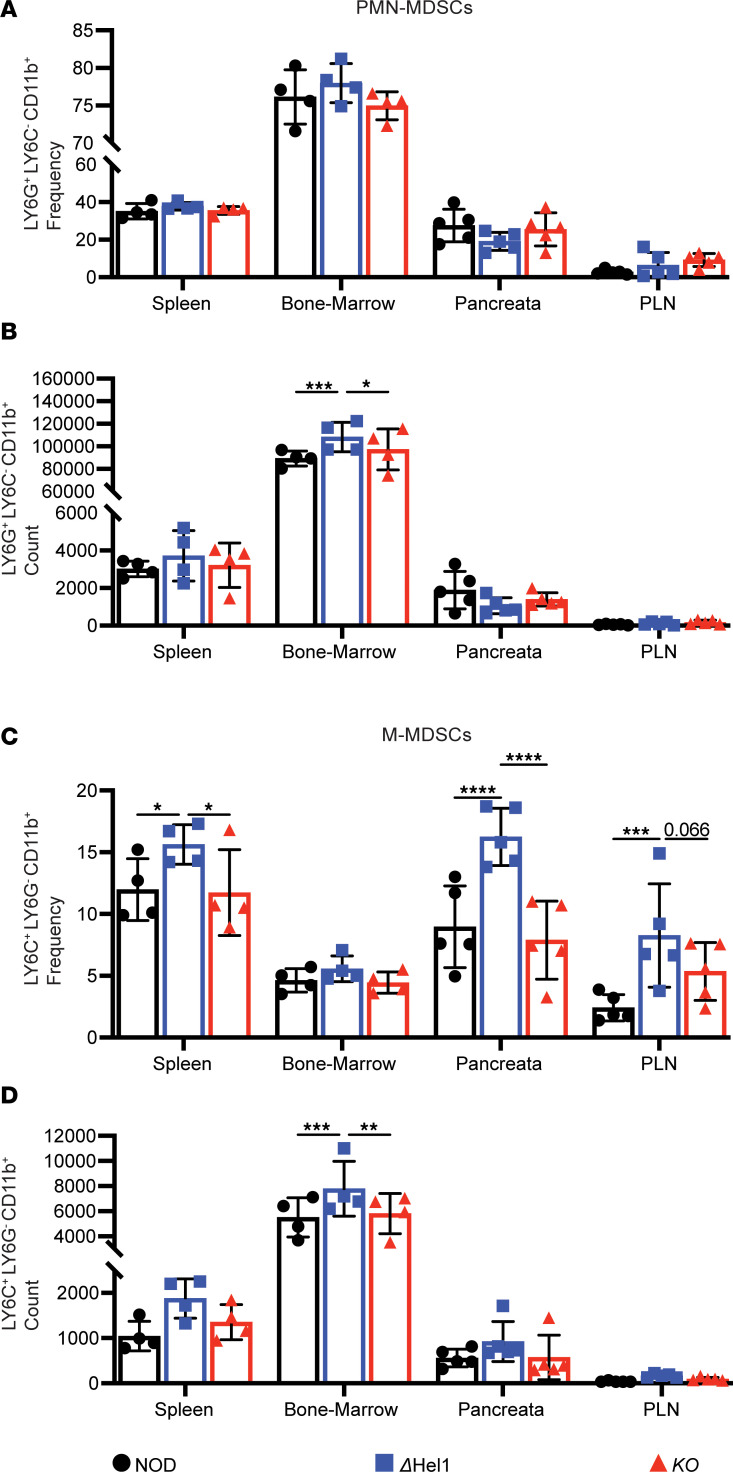
*Ifih1^ΔHel1^* mutation enhances MDSC populations. Flow cytometry analysis of LY6G^+^LY6C^–^CD11b^+^ neutrophil-like (PMN-MDSC) frequency (**A**) and cell count (**B**) and LY6C^+^LY6G^–^CD11b^+^ monocyte-like (M-MDSC) frequency (**C**) and cell count (**D**) of NOD, *ΔHel1*, and *KO* mice at 12 weeks of age. Analyzed by 2-way ANOVA with Tukey’s multiple comparisons (**A**–**D**). *P < 0.05, **P < 0.01, ***P < 0.001, ****P < 0.0001. *n* = 4–5.

Since *ΔHel1* mice have delayed T1D, but *KO* mice still develop T1D as do NOD mice, we investigated if MDSC populations were enhanced in *ΔHel1* mice or dampened in *KO* mice. We analyzed MDSCs in the spleen, bone marrow, pancreata, and PLNs of 12-week-old NOD, *ΔHel1*, and *KO* female mice during spontaneous T1D. The frequency of PMN-MDSCs was not different (Figure 4A), but cell counts were significantly increased within *ΔHel1* bone marrow compared with NOD (≈1.2-fold, *P* < 0.001) and *KO* mice (≈1.1-fold, *P* < 0.05) (Figure 4B). M-MDSC frequency was also increased in the *ΔHel1* spleen (≈1.3-fold, *P* < 0.05), pancreata (≈1.8-fold, *P* < 0.0001), and PLNs (≈3.4-fold, *P* < 0.001) compared with NOD (Figure 4C). The overall cell count of *ΔHel1* M-MDSCs was also increased within all organs but significantly increased in the bone marrow compared with NOD (≈1.4-fold, *P* < 0.001) and *KO* (≈1.3-fold, *P* < 0.01) mice (Figure 4D), whereas compared with *KO* mice, M-MDSC frequency was increased in the *ΔHel1* spleen (≈1.3-fold, *P* < 0.05), pancreata (≈2.1-fold, *P* < 0.0001), and PLNs (≈1.5-fold, *P* = 0.066) (Figure 4C). These results suggest that the *Ifih1*^ΔHel1^ mutation enhances MDSC populations, which may partially explain the delay in T1D development (Figure 1).

Since *KO* mice were not protected from T1D similar to NOD mice, loss of MDA5 may not promote MDSC development or may lead to MDSC deficiencies. We found no differences in PMN-MDSC or M-MDSC count or frequency in the spleen, bone marrow, pancreata, or PLNs of *KO* and NOD mice (Figure 4). These findings corroborate prior studies that type I IFN/IFNAR signaling is necessary for MDSC differentiation (43) and may partly explain how the loss of MDA5 expression in *KO* mice does not delay T1D (Figure 1).

### Ifih1^ΔHel1^ mutation leads to reduced MDA5 expression following MDA5-specific stimulation.

To verify that the *Ifih1^KO^* mutation resulted in a truncated form of or loss in MDA5 expression, we used an MDA5 antibody with specificity for AA 1–205 of the CARD in MDA5. Western blot analysis of MDA5 was detected in bone marrow–derived macrophages (BMDMs) from NOD and *ΔHel1* BMDMs stimulated with low–molecular weight poly(I:C), but MDA5 expression was absent in *KO* BMDMs (Supplemental Figure 4).

**Figure 5:**
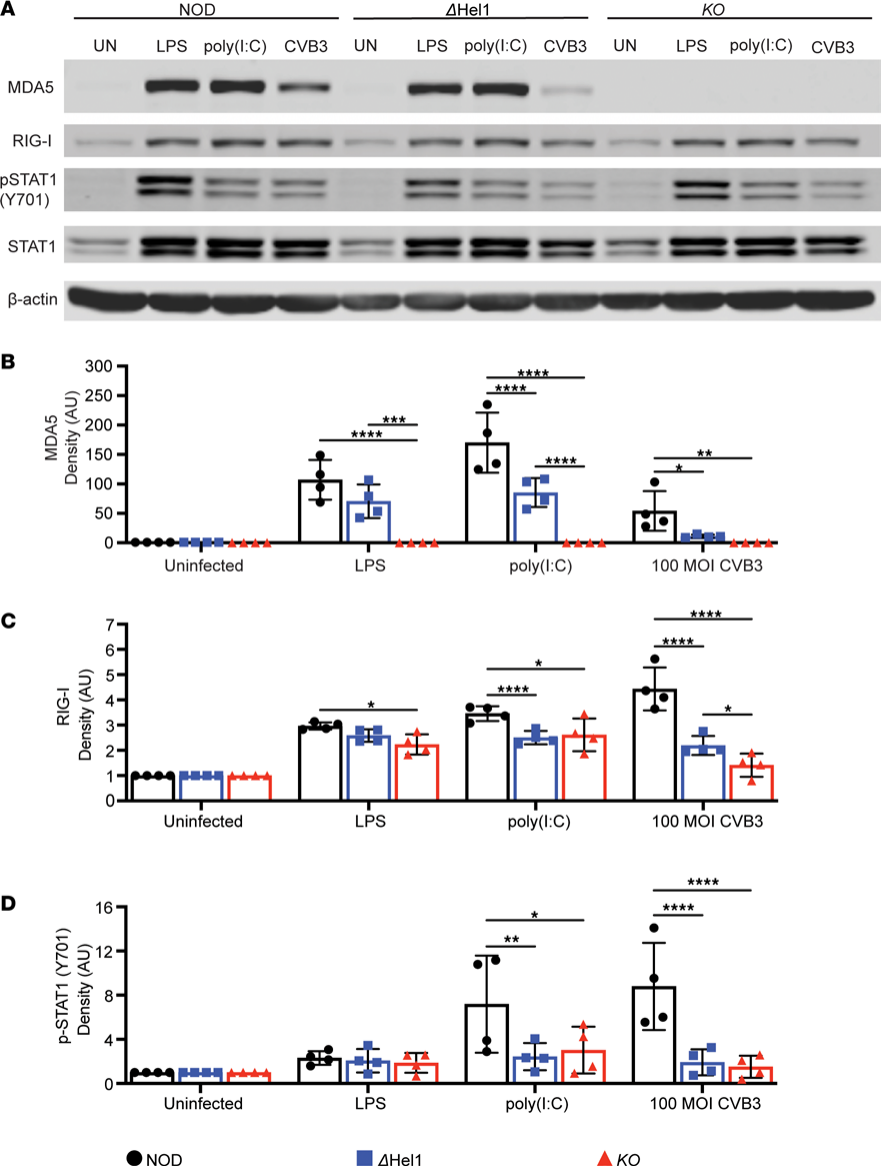
*Ifih1^ΔHel1^* mutation leads to reduced MDA5 expression following MDA5-specific stimulation. Western blot analysis of NOD, *ΔHel1*, and *KO* bone marrow–derived macrophages (BMDMs) stimulated with LPS, transfected HMW poly(I:C), and CVB3 for MDA5, RIG-I, p-STAT1 (Y701), and total STAT1 expression (**A**). p-, phosphorylated; RIG-I, retinoic acid-inducible gene I. Densitometry of MDA5 (**B**), RIG-I (**C**), and p-STAT1 (**D**) expression in BMDMs following stimulation. MDA5 and RIG-I are normalized to β-actin and to their respective unstimulated groups. P-STAT1 (Y701) is normalized to total STAT1, to β-actin, and to their respective unstimulated groups. Analyzed by 2-way ANOVA with Tukey’s multiple comparisons (**B**–**D**). *P < 0.05, **P < 0.01, ***P < 0.001, ****P < 0.0001. *n* = 4.

To examine the effect of *Ifih1* mutations on MDA5 expression in macrophages, we stimulated BMDMs from NOD, *ΔHel1*, and *KO* mice with lipopolysaccharide (LPS), transfected high–molecular weight (HMW) poly(I:C), or CVB3. Western blot analysis of MDA5 showed that stimulation of BMDMs from NOD mice with LPS, HMW poly(I:C), or CVB3 increased MDA5 expression (Figure 5, A and B). However, BMDMs from *ΔHel1* mice had a significant reduction in MDA5 expression after stimulation with HMW poly(I:C) (≈2.0-fold; *P* < 0.0001) and CVB3 (≈4.8-fold; *P* < 0.05) compared with NOD, but no differences were observed following stimulation with LPS. BMDMs from *KO* mice had no detectable MDA5 protein expression before or after stimulation (Figure 5, A and B). Reduced MDA5 expression from *ΔHel1* BMDMs, compared with NOD, suggest MDA5 responses and type I IFN synthesis may act as a positive feedback mechanism to further upregulate MDA5 expression (47, 48).

RIG-I expression was significantly downregulated in BMDMs from *ΔHel1* (≈2.0-fold, *P* < 0.05 and ≈1.4-fold, *P* < 0.0001) and *KO* (≈3.1-fold, *P* < 0.01 and ≈1.4-fold, *P* < 0.0001) mice following stimulation with HMW poly(I:C) and CVB3, respectively, compared with BMDMs from NOD mice (Figure 5, A and C). See complete unedited blots in the supplemental material. We also detected p-STAT1 (Y701) expression and observed a significant downregulation in BMDMs from *ΔHel1* (≈3.0-fold, *P* < 0.01 and ≈4.6-fold, *P* < 0.0001) and *KO* (≈2.4-fold, *P* < 0.05 and ≈5.9-fold, *P* < 0.0001) mice following stimulation with HMW poly(I:C) and CVB3, respectively, compared with NOD (Figure 5, A and D). Reduced RIG-I and p-STAT1 (Y701) expression in BMDMs from both *ΔHel1* and *KO* mice following MDA5 stimulation, compared with NOD, indicates that decreased MDA5 expression dampens type I IFN–mediated responses (49).

### ΔHel1 mice have improved viral clearance and reduced pancreatic IFN-α and IFN-β levels postinfection.

**Figure 6:**
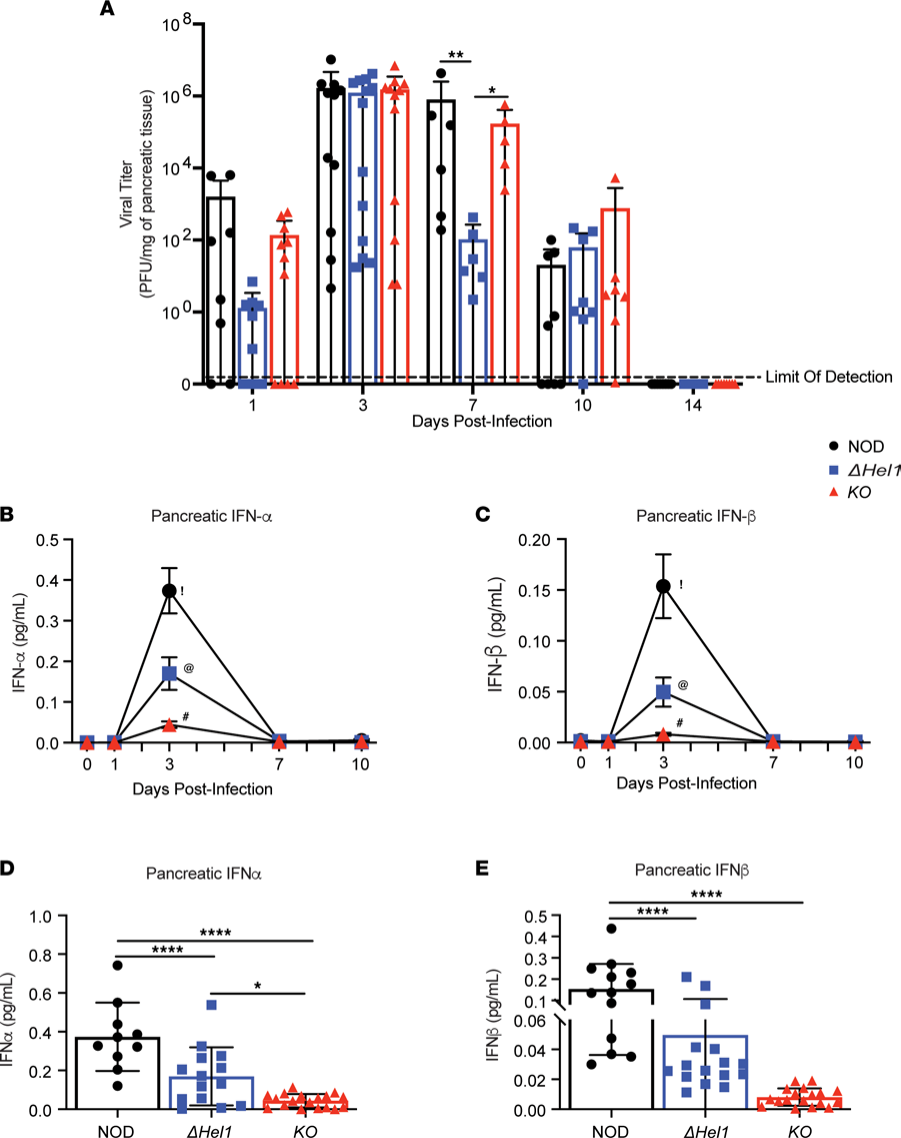
*ΔHel1* mice have improved viral clearance and reduced pancreatic IFN-α and IFN-β levels postinfection. Plaque assay of pancreatic CVB3 viral titer at days 1, 3, 7, 10, and 14 postinfection (**A**). ELISA analysis of pancreatic IFN-α (**B**) and IFN-β (**C**) levels at days 0, 1, 3, 7, and 10 following CVB3 infection of NOD, *ΔHel1*, and *KO* mice. ELISA analysis of pancreatic IFN-α (**D**) and IFN-β (**E**) concentrations at day 3 following CVB3 infection. Analyzed by 2-way ANOVA with Tukey’s multiple comparisons (**A**). Analyzed by 1-way ANOVA with Tukey’s multiple comparisons (**B**–**E**). *P < 0.05, **P < 0.01, ****P < 0.0001. ^!^ vs. ^@^ *P* < 0.0001, ^!^ vs. ^#^ *P* < 0.0001, ^@^ vs. ^#^ *P* < 0.05 (**B**). ^!^ vs. ^@^ *P* < 0.0001, ^!^ vs. ^#^ *P* < 0.0001 (**C**). (**A**) *n* = 5–13, and (**B**–**E**) *n* = 10–18.

To evaluate the effects of the *Ifih1* mutations on antiviral responses, we infected 12-week-old female and male NOD, *ΔHel1*, and *KO* mice with 100 PFU of CVB3/Woodruff and monitored viral clearance and pancreatic type I IFN production. Following CVB3 infection, viral clearance was determined by pancreatic viral titer on days 1, 3, 7, 10, and 14 postinfection in NOD, *ΔHel1*, and *KO* mice. Peak CVB3 pancreatic viral titer was observed on day 3 postinfection within all mice, but *ΔHel1* mice demonstrated a significant reduction on day 7 postinfection compared with both NOD (≈7,636-fold, *P* < 0.01) and *KO* (≈1,637-fold, *P* < 0.05) mice (Figure 6A). Corroborating the increase in viral titer, pancreatic IFN-α and IFN-β levels were maximal at day 3 postinfection and returned to basal levels by day 7 postinfection in all mice (Figure 6, B and C). At day 3 postinfection, CVB3-infected *ΔHel1* mice had a significant ≈2.2-fold (*P* < 0.0001) reduction in pancreatic IFN-α (Figure 6D) and ≈3-fold (*P* < 0.0001) decrease in IFN-β (Figure 6E) compared with NOD mice. CVB3-infected *KO* mice had a significant ≈8.5-fold (*P* < 0.0001) and ≈3.9-fold (*P* < 0.05) reduction in pancreatic IFN-α compared with NOD and *ΔHel1* mice, respectively (Figure 6D). *KO* IFN-β levels were also significantly reduced by ≈19.2-fold (*P* < 0.0001) compared with NOD mice, but no significant differences were observed between *ΔHel1* and *KO* (Figure 6E). The delay in CVB3-accelerated T1D observed in *ΔHel1* mice may be due to the optimal synthesis of type I IFNs necessary for viral clearance without inducing pancreatic inflammation and autoimmune activation. However, *KO* mice fail to produce robust levels of type I IFNs in response to CVB3 infection, which may contribute to CVB3-accelerated T1D without impairing viral clearance.

### Ifih1^ΔHel1^ mutation reduces MDA5-mediated ATP hydrolysis.

**Figure 7:**
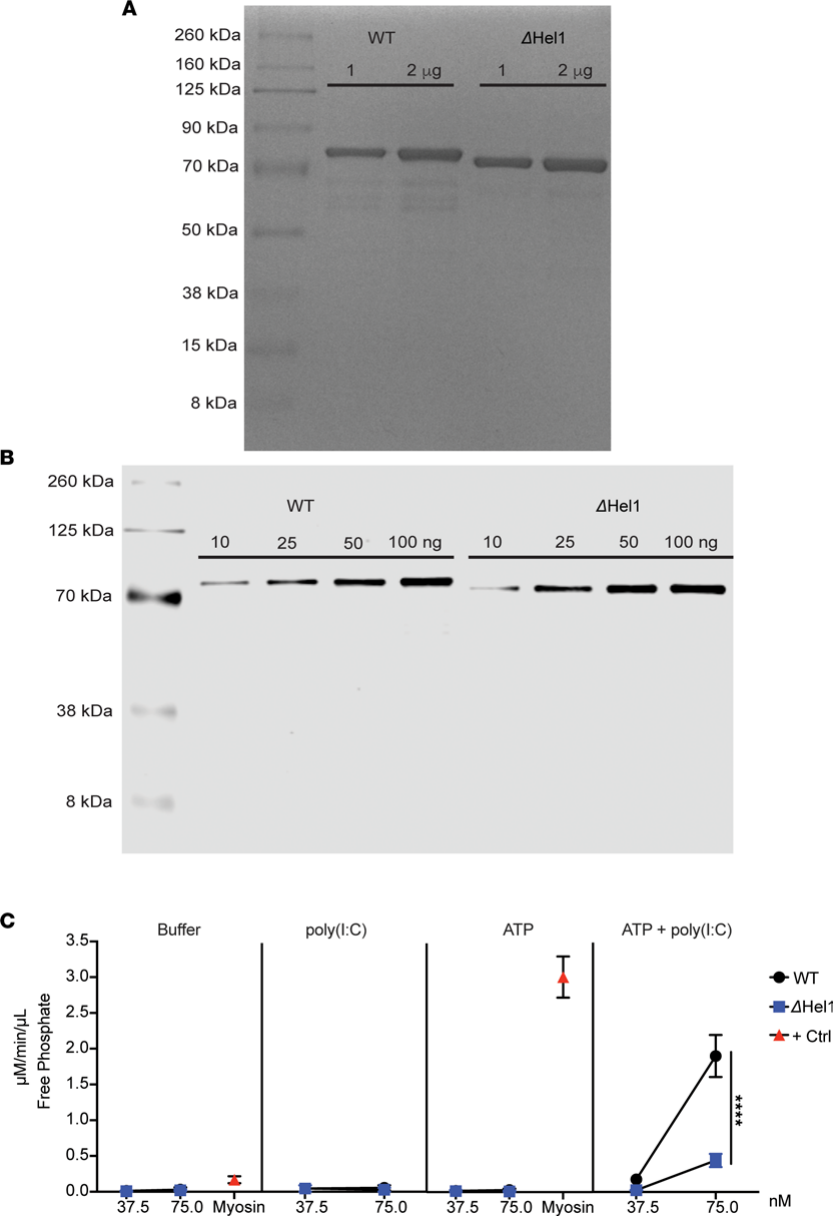
*Ifih1^ΔHel1^* mutation reduces MDA5-mediated ATP hydrolysis. Representative GelCode Blue staining of 1 and 2 μg of purified WT and *ΔHel1* MDA5 protein (**A**). Representative Western blot of 10, 25, 50, and 100 ng of purified WT and *ΔHel1* MDA5 protein (**B**). ATPase activity of WT and *ΔHel1* MDA5 protein at concentrations of 37.5 and 75.0 nM; 1,800 nM of myosin was used as a positive control to confirm ATP hydrolysis (**C**). Analyzed by 2-way ANOVA with uncorrected Fisher’s least significant differences (LSD) (**C**). ****P < 0.0001. (**A** and **B**) *n* = 2 and (**C**) *n* = 4.

MDA5 ATPase activity has been suggested to be a critical step required for MDA5 filament formation and disassembly, as well as its ability to interact with mitochondrial antiviral signaling protein (MAVS), which is required for downstream antiviral responses and type I IFN synthesis (31, 50, 51). Since the *Ifih1*^ΔHel1^ mutation is within an ATPase motif of the helicase 1 domain of MDA5 and leads to reduced type I IFN synthesis (Figure 6, B–E), we hypothesized that reduced *ΔHel1* immune responses and delayed T1D may be partly due to dampened ATPase activity in MDA5. We purified core WT and *ΔHel1* MDA5 protein without CARDs to measure ATP hydrolysis. To determine the purity of our MDA5 samples, we separated our purified fractions by SDS-PAGE and stained the gel with GelCode Blue. Coomassie staining of WT and *ΔHel1* MDA5 proteins revealed a prominent band around 83 kDa, with *ΔHel1* MDA5 having a reduced molecular mass (Figure 7A), consistent with the predicted molecular weight, 83.0 and 82.6 kDa, respectively. Our protein samples were also probed for MDA5 by Western blot, and a specific band for MDA5 around 83 kDa was detected (Figure 7B). See complete unedited blots in the supplemental material. Utilizing our purified WT and *ΔHel1* MDA5 protein in an ATPase assay, we observed that *ΔHel1* MDA5 protein was functional, but had a significant (≈4.3-fold, *P* < 0.0001) reduction in ATP hydrolysis following poly(I:C) stimulation, compared with WT MDA5 protein (Figure 7C). Collectively, these findings indicate that dampened MDA5 ATPase activity in the *ΔHel1* mouse may partly explain reduced proinflammatory immune cell responses and a delay in both spontaneous and CVB3-accelerated T1D.

## Discussion

CVB, mumps, rubella, and cytomegalovirus infections are linked to T1D development (52–57). Many studies focused on the link between CVB and T1D indicate that CVB may accelerate T1D development by inducing proinflammatory MDA5-dependent antiviral responses and bystander activation of T cells (58–60). *IFIH1* SNPs are associated with T1D (30), but how *IFIH1* mutations affect diabetogenicity is poorly understood. To investigate the role of MDA5 in T1D, we generated an in-frame deletion within the helicase 1 domain of MDA5 (*Ifih1*^ΔHel1^) and an out-of-frame deletion (*Ifih1^KO^*) in NOD mice with zinc finger nuclease–mediated (ZFN-mediated) gene targeting (61). The human T1D protective *IFIH1* alleles are associated with lower MDA5 expression or activity (27, 32, 62, 63) To mimic this, we investigated if the complete absence of MDA5 expression or its reduced activity can similarly confer T1D protection.

The *Ifih1*^ΔHel1^ mutation delayed both uninfected/spontaneous and CVB3-accelerated T1D, which was partly due to reductions in MDA5-mediated ATP hydrolysis, IFN-α/β synthesis, TNF^+^ macrophages, IFN-γ^+^CD4^+^ T cells, and perforin^+^CD8^+^ T cells in the pancreata. Therefore, decreased MDA5 function can reduce proinflammatory effector responses in T1D. However, the *Ifih1^KO^* mutation did not delay spontaneous or CVB3-accelerated T1D in female mice and, surprisingly, enhanced spontaneous T1D in male mice. Uninfected *KO* mice exhibited no reduction in pancreatic TNF^+^ macrophages, IFN-γ^+^CD4^+^ T cells, and perforin^+^CD8^+^ T cells during spontaneous T1D. While CVB3-infected *KO* mice had fewer pancreatic IFN-γ^+^CD4^+^ and perforin^+^CD8^+^ T cells compared with NOD mice, TNF^+^ macrophages were still elevated. However, during spontaneous T1D development, *ΔHel1* mice had increased MDSC populations compared with NOD and *KO*, but no alteration in MDSCs was observed in *KO* mice compared to NOD. Failure to increase MDSCs in *KO* mice may partly explain their susceptibility to developing T1D similar to NOD mice.

Our results demonstrated that MDA5-dependent responses can dictate T1D progression partly mediated by pancreas-infiltrating macrophages. Macrophages regulate inflammatory responses in the islet, facilitate T cell recruitment, and activate autoreactive T cells (64, 65). NOD and *KO* mice had robust proinflammatory pancreatic macrophages during spontaneous and CVB3-accelerated T1D, but TNF^+^ macrophage populations were reduced in *ΔHel1* mice. *ΔHel1* pancreatic macrophages are inherently less inflammatory, possibly preventing β cell destruction and T1D.

Effector CD4^+^ and CD8^+^ T cells mediate β cell destruction and T1D development (66). During spontaneous T1D development, *ΔHel1* mice had reduced numbers of pancreatic IFN-γ^+^CD4^+^ and perforin^+^CD8^+^ T cells compared with NOD and *KO* mice, which may partly explain the delay in T1D progression. However, both *ΔHel1* and *KO* mice had reduced numbers of IFN-γ^+^CD4^+^ and perforin^+^CD8^+^ T cells compared with NOD following CVB3 infection. These findings provide evidence that MDA5-dependent type I IFN synthesis is necessary for maturing CD4^+^ and CD8^+^ T cell effector responses during CVB3-accelerated T1D. The decrease in type I IFN synthesis in the pancreata of CVB3-infected *ΔHel1* mice may not be sufficient for maturing CD4^+^ and CD8^+^ T cell antiviral effector responses, thereby delaying CVB3-accelerated T1D.

Interestingly, *KO* mice still developed spontaneous and CVB3-accelerated T1D but failed to produce heightened levels of type I IFNs within the pancreata following infection. The development of spontaneous T1D in *KO* mice may be due to an increase in proinflammatory pancreatic macrophages and effector T cells and, concomitantly, reduced Treg and/or MDSC populations. Our results with the *KO* mouse show that MDA5-mediated type I IFNs may be necessary to prevent T1D. Robust type I IFN synthesis during viral infections inhibits Treg activation and proliferation, but a complete loss of type I IFN signaling in Tregs impairs Treg FoxP3 expression and suppressor function (67, 68). Although we observed no difference in pancreatic Treg populations between NOD, *ΔHel1*, and *KO* mice, there was a marked reduction in Tregs from the PLNs of *KO* mice compared with *ΔHel1* (Supplemental Figure 3). Whether loss of MDA5 in *KO* mice impairs Treg suppressive function due to diminished type I IFN signaling needs to be determined.

Another immunosuppressive immune cell type that may be influenced by MDA5-dependent signals is MDSCs. These cells produce immunomodulatory molecules that dampen inflammatory immune responses (38). Impaired MDSC function has been linked to human T1D (42), and adoptive transfer of MDSCs can delay T1D in NOD mice (41). In pancreatic cancer models, type I IFNs play an important role in regulating MDSC suppressor activity. Too much or too little type I IFN signaling is detrimental to MDSC suppressor function, but an intermediate amount of type I IFN signaling may result in optimal MDSC function (43–45). These reports support the differences we observed in uninfected/spontaneous and CVB3-accelerated T1D with *ΔHel1* and *KO* mice and provide evidence that altered type I IFN signaling may affect MDSC function.

We observed an increase in MDSCs in *ΔHel1* mice within multiple organs, including the spleen, bone marrow, pancreata, and PLNs. These findings suggest that MDSCs in *ΔHel1* mice may suppress inflammatory pancreatic macrophage and T cell responses, thereby delaying both spontaneous and CVB3-accelerated T1D. During spontaneous T1D, MDSC populations between NOD and *KO* mice were comparable and may partly explain why *KO* mice still develop autoimmune diabetes similar to NOD mice. It remains plausible that NOD mice lose MDSC suppressor function during spontaneous T1D and the absence of MDA5-dependent type I IFN synthesis can contribute to a loss in MDSC suppressor function as observed in our *KO* model. Future studies will examine if MDSCs or Tregs from *ΔHel1* and *KO* mice exhibit enhanced or defective immunosuppressive function, respectively, in contrast to NOD mice.

Our *ΔHel1* mouse validated and expanded upon previous findings by Lincez et al. with NOD.MDA5^+/–^ mice, which had delayed spontaneous and CVB4-accelerated T1D due to decreased MDA5 expression, had reduced *Ifna* mRNA, had a dampened CD4^+^ T cell effector response, and had a concomitant increase in Treg populations in the PLNs (13). Although we observed no differences in Treg populations in NOD, *ΔHel1*, and *KO* mice, it is plausible that *ΔHel1* mice may exhibit an increase in Treg suppressive function without affecting Treg numbers. Nevertheless, our 2 independent studies using different coxsackievirus strains and mouse models highlight the importance of MDA5-dependent responses in T1D.

Mutations in *IFIH1* are also associated with other autoimmune diseases, including multiple sclerosis (MS) (69, 70), systemic lupus erythematosus (SLE) (71, 72), and rheumatoid arthritis (RA) (73, 74). Therefore, it is important to define how genetic mutations in *IFIH1* contribute to MDA5 function, since this knowledge would apply not only to T1D but also to other autoimmune diseases. Patients with SLE and with an MDA5 gain-of-function mutation R779H (rs587777446) have increased IFN-α serum levels (75), which may be due to dysregulated helicase ATP hydrolysis and dsRNA binding (76). The A946T and R843H SNPs within *IFIH1* are associated with T1D risk. The A946T SNP results in increased MDA5 function and type I IFN synthesis, but the effect of the R843H SNP (rs3747517) remains unknown (30).

Conversely, some *IFIH1* SNPs, such as E627x and I923V, are associated with protection (30). The E627x SNP leads to reduced MDA5 expression and type I IFN synthesis due to a premature stop codon (32). The I923V SNP also has reduced type I IFN synthesis, but this appears to be due to I923V MDA5 forming shorter filaments, having decreased ATP hydrolysis and enhanced dsRNA dissociation (27, 31). The protective effect of E627x and I923V SNPs in *IFIH1* appears to be due to either reduced MDA5 expression or reduced ATP hydrolysis and subsequently, diminished type I IFN synthesis.

Gorman et al. showed that overexpression of the A946T SNP causes increased *IFNB1* expression, while the I923V SNP leads to reduced expression of *IFNB1* (27). It remains unclear if the A946T SNP promotes T1D due to increased basal MDA5 activity (27) or altered response to ligands, such as self-dsRNA, CVB3, or endogenous retroelements (77–79). Nevertheless, MDA5 activity and signaling may be a key driving factor in T1D development by promoting type I IFN synthesis. Further investigation is warranted to determine whether mutations in MDA5 results in abnormal ligand binding and/or basal MDA5 activity leading to autoimmunity.

ATPase activity has been shown to tightly regulate the stability of MDA5 during filament formation and disassembly in response to dsRNA (31) and is critical to prevent MDA5 binding to self-RNA (80). MDA5 models indicate that regulation of MDA5 disassembly by ATP hydrolysis may be required for MDA5-dependent interaction with MAVS and subsequent antiviral signaling (31, 50, 51). The *Ifih1*^ΔHel1^ mutation is located within an ATPase motif in the helicase 1 domain of MDA5. *ΔHel1* MDA5 protein has functional ATPase activity following poly(I:C) stimulation, but compared with WT MDA5 protein, ATP hydrolysis was reduced, which may result in dampened signaling downstream of MDA5. A subsequent effect of reduced MDA5 function appears to be reduced type I IFN synthesis and immune cell activation in pancreata, leading to a delay in spontaneous and CVB3-accelerated T1D. Our results may parallel the phenotype of dampened ATP hydrolysis in the I923V SNP, thereby reducing type I IFN synthesis and delaying T1D development.

Our study may provide the rationale for the development of small molecule inhibitors that target the ATPase motifs within the helicase domains of MDA5 to reduce type I IFN synthesis. This potentially novel therapeutic approach may dampen autoreactive T cell responses, decrease β cell damage, and delay T1D development similar to the protection observed in *ΔHel1* mice. Future translational studies are warranted to assess whether alterations of the helicase 1 domain in human MDA5 can affect innate and adaptive immune responses and, subsequently, elicit immunoprotection against autoimmune diabetes. In addition to T1D, MDA5 inhibitors may also be useful in the treatment of other autoimmune diseases where exacerbated MDA5 responses may play a role, such as SLE (71, 72), MS (69, 70), or RA (73, 74).

## Methods

### Animals.

NOD/ShiLtJ (NOD) and NOD.*Rag* mice were purchased from The Jackson Laboratory, while NOD.*Ifih1*^ΔHel1^ (*ΔHel1*) and NOD.*Ifih1^KO^* (*KO*) mice were provided by the Medical College of Wisconsin, Department of Pediatrics. All mice were bred and housed under pathogen-free conditions in the Research Support Building animal facility at The University of Alabama at Birmingham. *ΔHel1* and *KO* mice were generated by ZFN-mediated gene targeting as described (61). Constructs of the ZFN pairs specifically targeting exon 6 of the mouse *Ifih1* gene were designed, assembled, and validated by MilliporeSigma (target sequence ATCTGGAGAGTGGAGAcgatgACGGTGTGCAGCTGTCAGG; ZFNs bind to each sequence shown in uppercase on opposite strands). mRNAs encoding ZFN pairs were prepared in injection buffer (1 mM Tris-HCl, 0.1 mM EDTA, pH 7.4) at a concentration between 5 and 10 ng/μL and injected into the pronucleus of fertilized NOD 1-cell embryos. Injected embryos were transferred to pseudopregnant CD-1 females (Charles River). Tail DNA was extracted and screened for ZFN-induced mutation by PCR amplification with forward (5′-TGGATTAAGTGGCGATACCC-3′) and reverse (5′-TTTTCAGGGAAGTGGAGCAC-3′) primers and standard sequencing. Identified founders were backcrossed to NOD mice followed by intercrossing to fix the mutation to homozygosity. Mice were maintained on a 12-hour light/12-hour dark cycle at 23°C and received standard lab chow and acidified water weekly.

### In vivo infections, diabetes incidence, and viral plaque assays.

NOD, *ΔHel1*, and *KO* male and female mice at 12 weeks of age were infected by i.p. injection with 100 PFU CVB3/Woodruff in HBSS or HBSS control as published (58). Diabetes incidence was monitored every other day by glucosuria (Diastix) and confirmed by 2 consecutive blood glucose readings ≥ 300 mg/dL with a Contour Next meter (Ascensia Diabetes Care) until 40 weeks of age. Pancreatic viral titers were performed as described (81).

### Insulitis scoring.

Pancreata from mice were fixed in 4% (*w/v*) paraformaldehyde dissolved in phosphate buffer (0.12 M; pH 7.4), processed, and embedded in paraffin. Pancreata were sectioned, then stained with hematoxylin and eosin, and insulitis scoring was performed as we published (82).

### IPGTT and GSIS.

Mice were fasted for 15 hours, followed by an intraperitoneal injection of 2 g/kg body weight sterile filtered 20% glucose solution in PBS. Blood glucose was measured at 0, 5, 15, 30, 60, 90, and 120 minutes postinjection as described above. GSIS was performed on islets from 12-week-old male NOD, *ΔHel1*, and *KO* mice as previously described (83).

### Flow cytometry.

Flow cytometry was performed on pancreatic cells from NOD, *ΔHel1*, and *KO* female mice at 12 weeks of age and following CVB3 infection as published (13). Pancreata were harvested in 2 mL of RPMI 1640 (Invitrogen, Thermo Fisher Scientific) with 300 U/mL of collagenase type 4 (Worthington, LS004188), digested in a 37 °C water bath for 15 minutes with agitation every 5 minutes, and homogenized with a Dounce homogenizer. Samples were treated with GolgiPlug (BD Biosciences) with or without 100 ng/mL PMA and 1 μg/mL ionomycin, Fc receptors were blocked, and surface or intracellular flow cytometry was performed with fluorochrome-conjugated antibodies as previously described (84) (Supplemental Table 1A). For intracellular staining, cells were fixed and permeabilized with eBioscience (Thermo Fisher Scientific) FoxP3 transcription factor fix/perm overnight. Cells were analyzed with an Attune NxT flow cytometer (Thermo Fisher Scientific) with ≈1,000,000 events/sample and analyzed with FlowJo version 10.6.2 software. The flow cytometry gating scheme is shown in Supplemental Figures 5 and 6. The average viability of pancreatic samples digested with collagenase was about 65%–80%, and any sample with less than 50% viability was excluded from analysis.

### BMDMs.

BMDMs were generated from NOD, *ΔHel1*, and *KO* male mice at 8–12 weeks of age as previously described (85). BMDMs were stimulated with 1 μg/mL of transfected HMW poly(I:C) using LyoVec (InvivoGen), 1 μg/mL LPS (*E*. *coli* 055:B5), or 100 MOI CVB3/Woodruff.

### Western blot and Coomassie staining.

MDA5, RIG-I, p-STAT1 (Y701), and STAT1 expression in untreated or poly(I:C)-, LPS-, or CVB3-stimulated BMDM whole-cell lysates was detected by Western blot analysis as previously described (86). Purified proteins from size-exclusion chromatography were separated by SDS-PAGE, Coomassie stained with GelCode Blue (Thermo Fisher Scientific), and then probed for MDA5 by Western blot analysis. Proteins were detected by incubation with primary antibodies (Supplemental Table 1B) followed by an anti-rabbit IRDye 680/800CW secondary antibody (LI-COR Biosciences), visualized on an Odyssey CLx Imager with Image Studio v4.0 software (LI-COR Biosciences) to calculate densitometry, and normalized to β-actin and unstimulated controls.

### Adoptive transfer of diabetes.

Sixteen-week-old nondiabetic female NOD, *ΔHel1*, and *KO* spleens were resuspended at 10^8^ cells/mL in HBSS. Ten million splenocytes were transferred intravenously into 10-week-old female NOD.*Rag* mice, which were monitored for diabetes as described above.

### IFN-α/β ELISA.

Pancreata were collected in 1 mL of PBS containing 14.29% of Protease Inhibitor Cocktail (Roche, MilliporeSigma; 11836153001), homogenized with an electric homogenizer, and centrifuged at 12,000 RCF for 10 minutes at 4°C. Supernatants were transferred to new tubes and frozen overnight. Samples were thawed, centrifuged (at 12,000 RCF for 10 minutes at 4°C), and used in an IFN-α and IFN-β ELISA (PBL Assay Science, 42120-2 and 42400-2) according to the manufacturer’s protocol. IFN-α and IFN-β levels were normalized to total protein measured by BCA assay (Thermo Fisher Scientific, 23227). Plates were read on a Synergy2 microplate reader, and the data were analyzed with Gen5 v.1.10 software (BioTek).

### Expression and purification of MDA5 protein.

Mouse MDA5 (*Ifih1*) with CARDs removed (AA 304–1025) was subcloned into the pET His TEV LIC vector (Addgene) with an N-terminal hexa-histidine tag and a tobacco etch virus (TEV) protease cleavage site (50, 51). The Hel2i L2 surface loop AA 646–663 was deleted to increase solubility (51). As previously shown, deletion of the L2 loop does not alter MDA5 ATPase activity, type I IFN signaling, or dsRNA binding (27, 51, 87, 88). The *ΔHel1* mutation was introduced by deleting AA 428–432, using a Q5 Site-Directed Mutagenesis Kit (New England Biolabs), and both WT and *ΔHel1* constructs were verified by DNA sequencing.

### E.

*coli* BL21 (DE3) cells were transformed with MDA5 constructs and grown to OD_600_ 0.6–0.8 absorbance units at 37°C, as described (51). The temperature was reduced to 18°C, and protein expression was induced with 0.25 mM isopropyl-β-D-1-thiogalactopyranoside (Fisher Scientific) overnight. Cells were harvested by centrifugation (6,200 RCF for 15 minutes at 4°C) and resuspended in 20 mM Tris, 0.5 M NaCl, and 5 mM imidazole at pH 7.9, then lysed by ultrasonication on ice. Lysates were spun at 27,000*g* for 45 minutes at 4°C, and supernatant was loaded onto Ni-NTA agarose (Thermo Fisher Scientific). Protein was washed with 50 mM HEPES at pH 7.5, 0.15 M NaCl, 5% glycerol, 20 mM imidazole, and 8 mM 2-mercaptoethanol (β-ME), and MDA5 was eluted with 50 mM HEPES pH 7.5, 0.15 M NaCl, 5% glycerol, 0.3 M imidazole, and 8 mM β-ME. MDA5 was then purified on a heparin column (GE) (buffer A: 20 mM HEPES pH 7.5, 0.1 M NaCl, 2 mM dithiothreitol [DTT]; buffer B: 20 mM HEPES pH 7.5, 1 M NaCl, 2 mM DTT), then a Superdex 200 10/300 GL size-exclusion column (GE) by NaCl gradient in buffer 20 mM HEPES pH 7.5 and 2 mM DTT. Final protein was purified by size-exclusion chromatography on a Superdex 200 10/300 GL column in 20 mM HEPES pH 7.5, 0.5M NaCl, 5 mM MgCl_2_, and 2 mM DTT, similar to methods described (51).

### ATPase activity assay.

ATP hydrolysis by MDA5 was measured by the Malachite green assay (MilliporeSigma, MAK113-1KT). Purified MDA5 at 37.5 and 75.0 nM was incubated with 4 μg/mL of HMW poly(I:C) (InvivoGen) and 4 mM ATP (MilliporeSigma, A6419) for 30 minutes at room temperature in assay buffer according to the manufacturer’s protocol. As a positive control for ATP hydrolysis, 1,800 nM of myosin (MilliporeSigma, M0531) was incubated with or without 4 mM ATP. Malachite green reagent was added and developed for 30 minutes at room temperature. Plates were read with a Synergy2 microplate reader and analyzed with Gen5 v.1.10 software.

### Statistics.

Data were analyzed using GraphPad Prism Version 8.0 statistical software. Statistical analysis between each experimental group was performed by 1-way ANOVA, 2-way ANOVA, or log-rank (Mantel-Cox) test, with Tukey’s multiple comparisons or uncorrected Fisher’s LSD as stated in the figure legends, with *P* < 0.05 considered significant. Error bars represent the SD of each data set. All experiments were performed independently at least 4 separate times with data obtained in a minimum of triplicate wells in each in vitro experiment.

### Study approval.

All animal studies were approved by the University of Alabama at Birmingham Institutional Animal Use and Care Committee and performed per the University of Alabama at Birmingham Institutional Animal Use and Care Committee mouse guidelines and the NIH’s *Guide for the Care and Use of Laboratory Animals* (National Academies Press, 2011).

## Author contributions

SIB, JPT, ARB, AMG, SQ, TJG, and YGC designed the research studies, conducted experiments, acquired data, analyzed data, and wrote the manuscript. QS, OS, and ARH conducted experiments. JMB conducted experiments, acquired data, and analyzed data. HMT designed the research studies, analyzed data, and wrote the manuscript. HMT is the guarantor of this work and, as such, had full access to all the data in the study and takes responsibility for the integrity of the data and the accuracy of the data analysis.

## Supplementary Material

Supplemental data

## Version 1 12/13/2022

In-Press Preview

## Version 2 01/24/2023

Electronic publication
