# Supplementary material for: MDA5-dependent responses contribute to autoimmune diabetes progression and hindrance
Source: JCI Insight. 2023 Jan 24;8(2):e157929. doi: 10.1172/jci.insight.157929 (PMC9977297; doi:10.1172/jci.insight.157929)

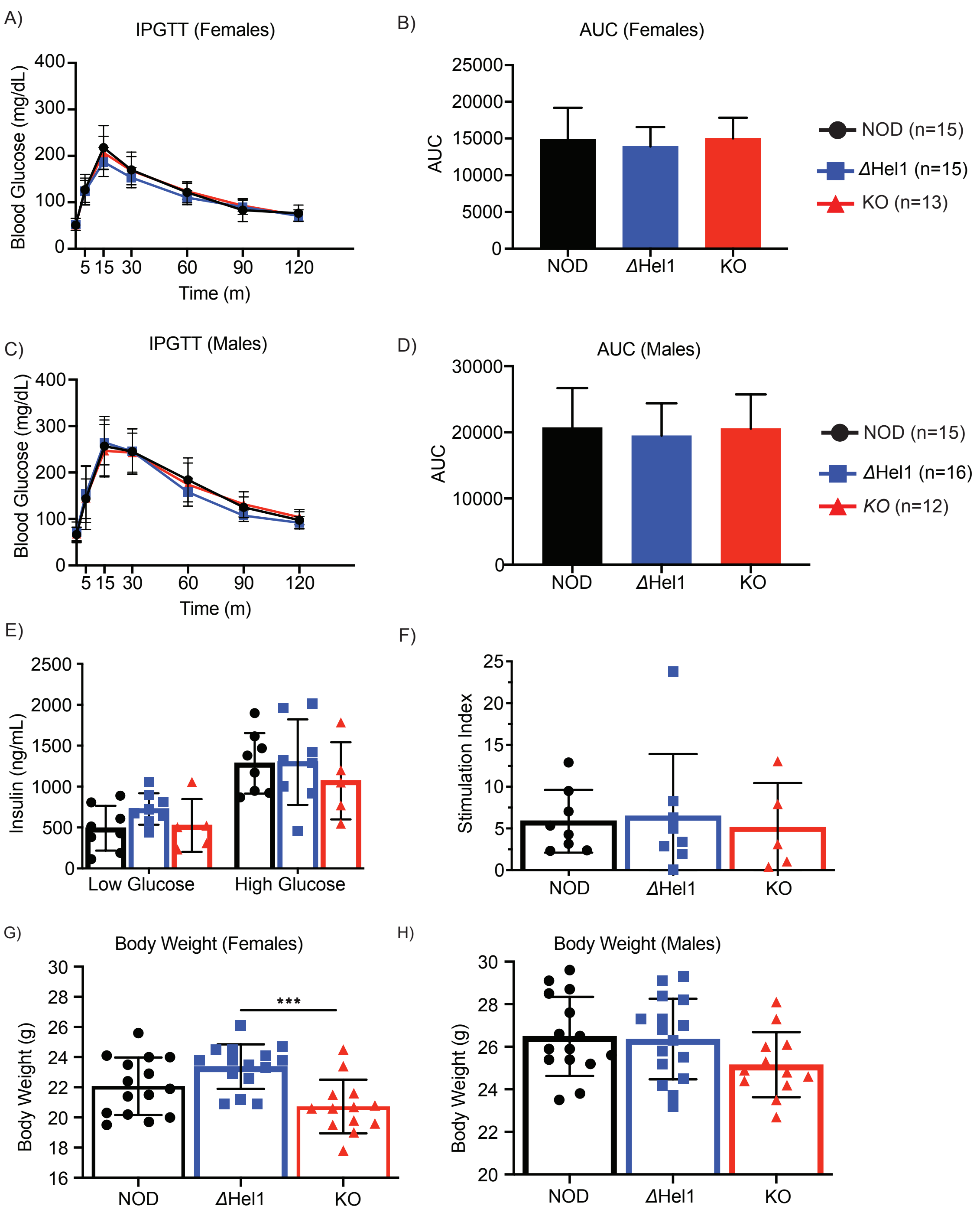

Supplemental Figure 1: IPGTT and GSIS of male and female NOD, *ΔHel1*, and *KO* mice.

Intraperitoneal glucose tolerance test of 12-week-old NOD, *ΔHel1*, and *KO* female mice (A, B) and male (C, D) mice with area under the curve (AUC) analysis. Glucose-stimulated insulin secretion from isolated NOD, *ΔHel1*, and *KO* islets (**E**) and insulin stimulation index normalized to total insulin content (F). Weightof 12-week-old NOD, *ΔHel1*, and *KO* female (G) and male (H) mice. Analyzed by one-way ANOVA with multiple comparisons (G). *** p<0.001. A, B, C, D: n=12-16; A, B, C, D: n=16-25, E, F: n=5-8 and G, H n=13-15.

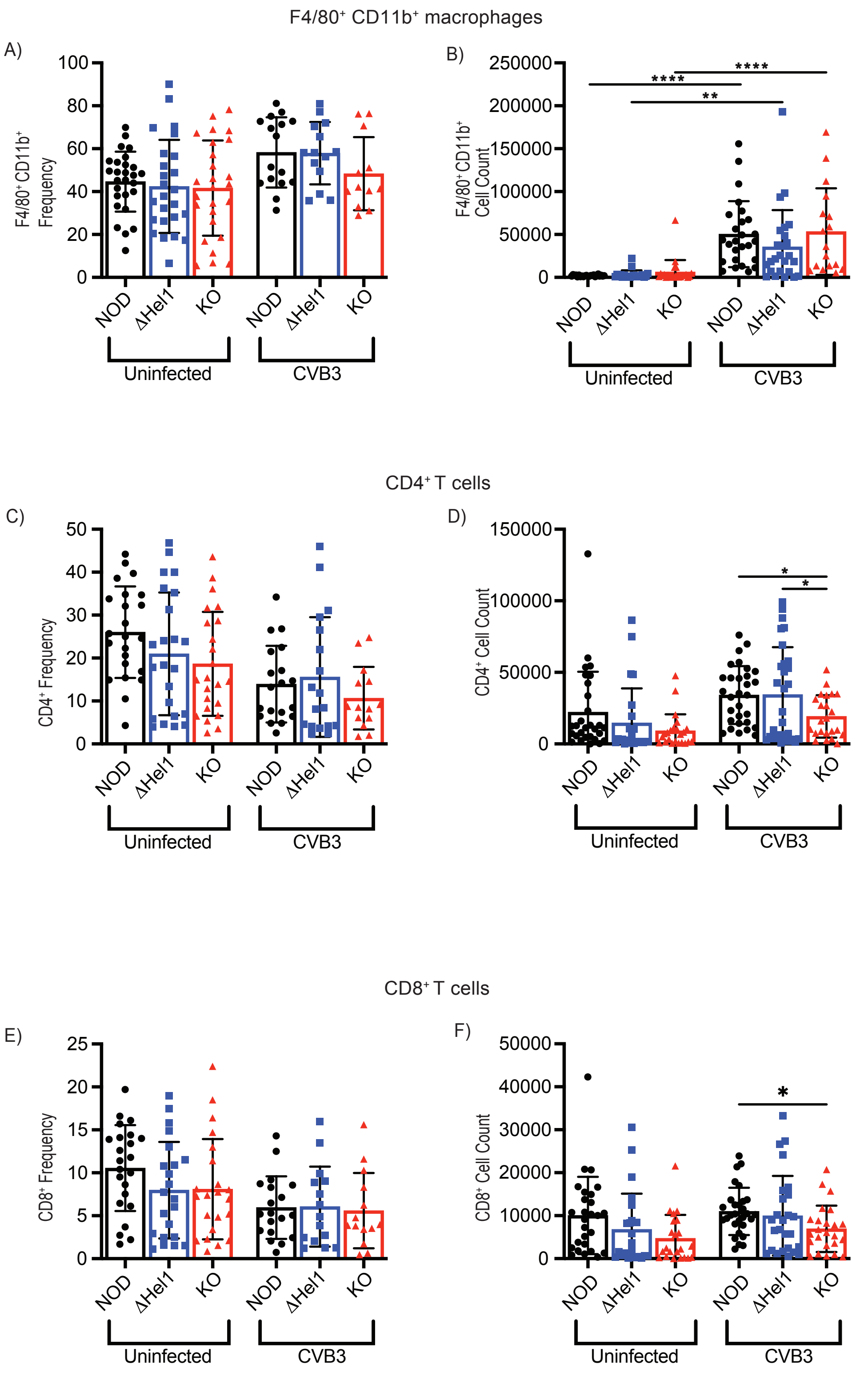

Supplemental Figure 2: Flow cytometry analysis of pancreatic macrophages, CD4 and CD8 T cells at day 7 post-CVB3 infection.

Flow cytometry analysis of pancreatic immune cells for F4/80⁺ CD11b⁺ frequency (A) and cell counts (B); CD4⁺ T cell frequency (C) and cell counts (D); CD8⁺ T cell frequency (E) and cell counts (F) Analyzed by two-way ANOVA with Tukey's multiple comparisons (B). ** $p<0.01$, *** $p<0.001$. A-F: n=16-25.

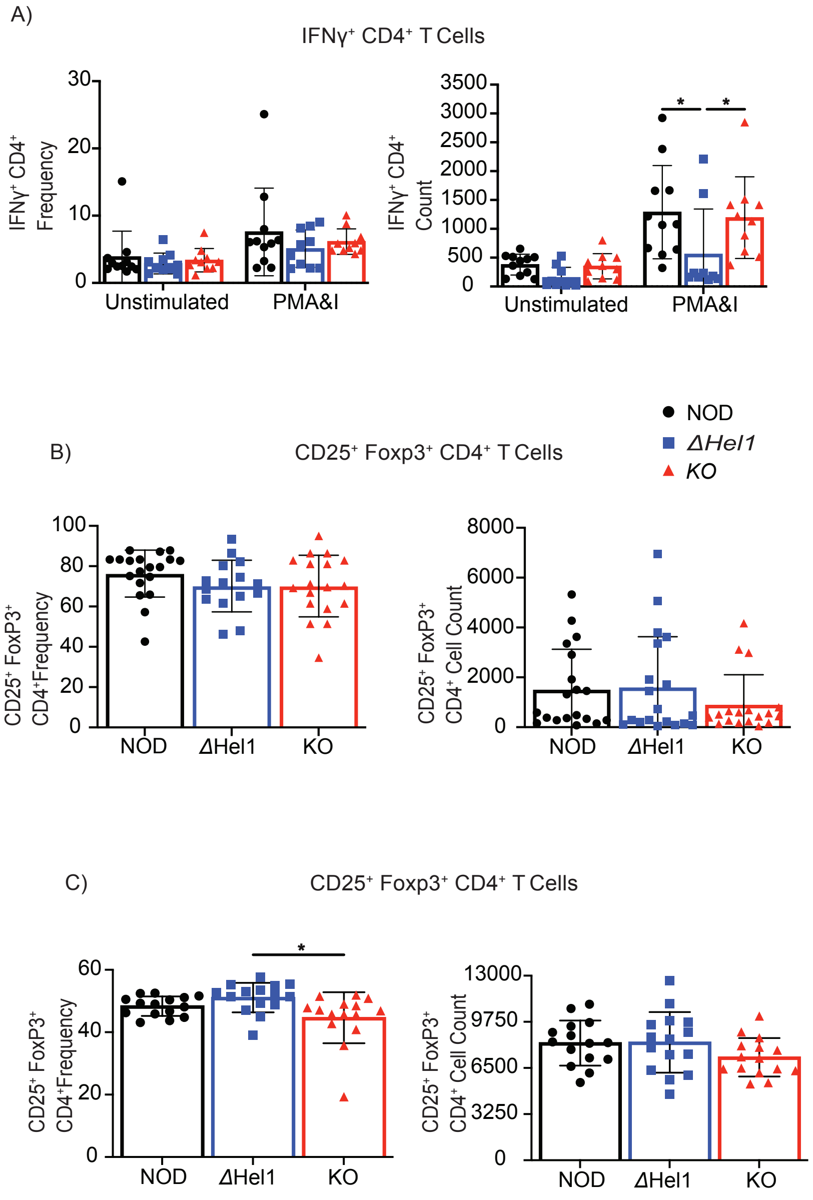

Supplemental Figure 3: Flow cytometry analysis of macrophage and T cell populations during spontaneous T1D.

Flow cytometry analysis of pancreatic IFNγ$^+$ CD4$^+$ T cells (A); CD25$^+$ FoxP3$^+$ CD4$^+$ T cells (B); and CD25$^+$ FoxP3$^+$ CD4$^+$ T cells from the pancreatic lymph node (C); frequency and cell counts of 12-week-old NOD, *ΔHel1,* and *KO* female mice. Analyzed by two-way ANOVA with Tukey's multiple comparisons (A), and analyzed by one-way ANOVA with Tukey's multiple comparisons (C). * $p<0.05$. A, B, C: n=16-25, D: n=9-11, E: n=15-19, and F: n=15.

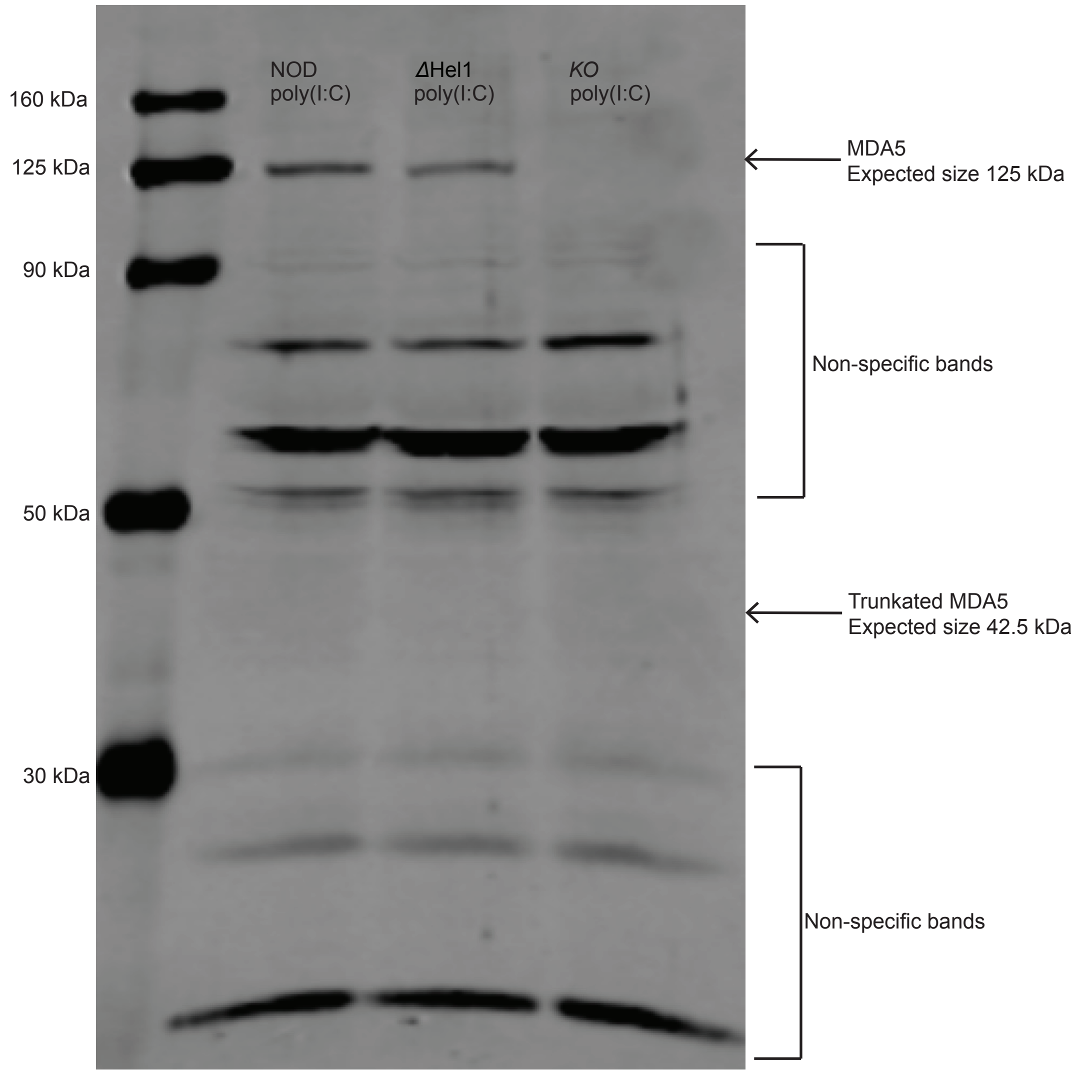

Supplemental Figure 4: KO mutation does not lead to the expression of truncated MDA5 protein.

Representative Western blot analysis of NOD, *ΔHel1*, and *KO* bone marrow-derived macrophages (BMDMs) stimulated with LMW poly(I:C) for MDA5. MDA5 was measured using Fisher Scientific MDA5 Polyclonal Antibody CN: PIPA5-89344, and 40 µg of total protein was used per sample. n=3

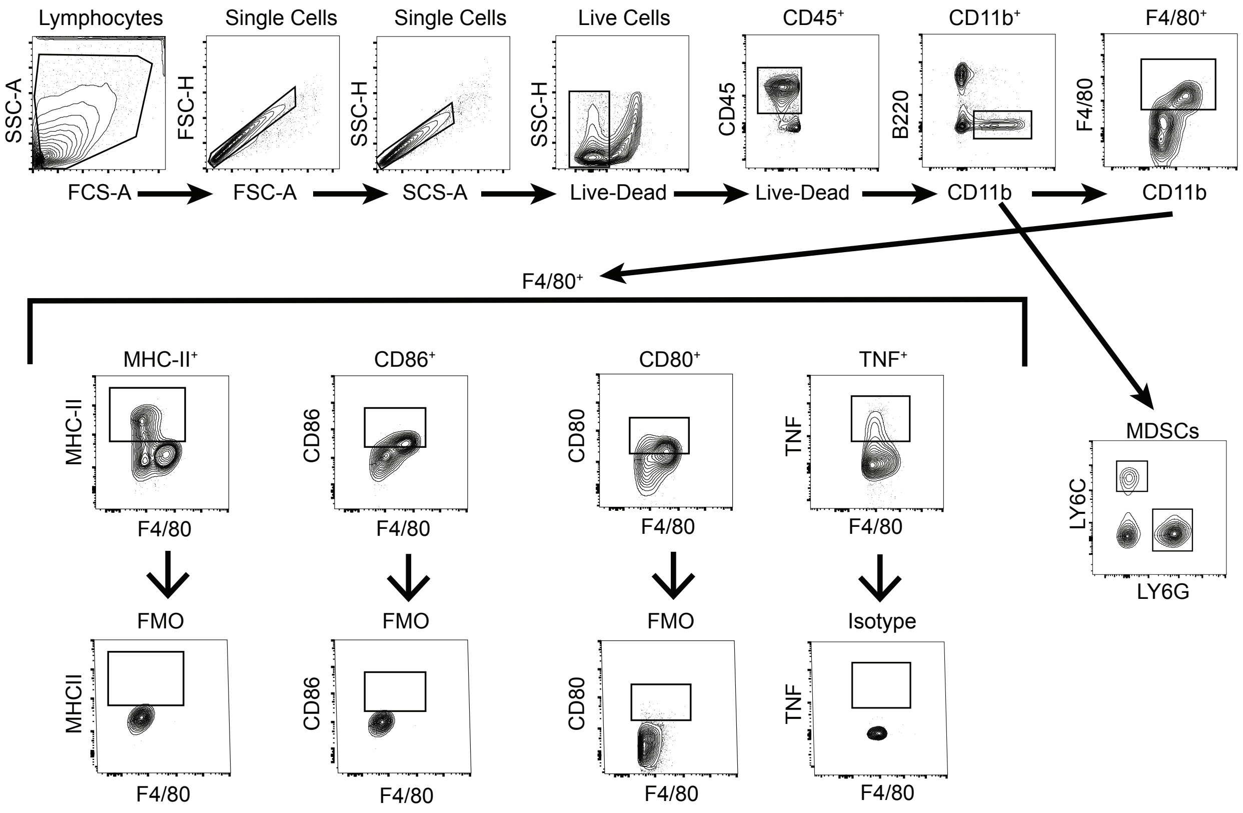

Supplemental Figure 5: Flow cytometry gating of innate immune cells.

Cells were pre-gated on single cells, live cells, and CD45⁺ cells for immunophenotyping of macrophages (CD11b, F480), PMN-MDSCs (CD11b, LY6G), and M-MDSCs (CD11b, LY6C). fluorescence minus one (FMO) is also shown for MHC-II, CD80, and CD86. Isotype is shown for intracellular cytokine staining for TNF.

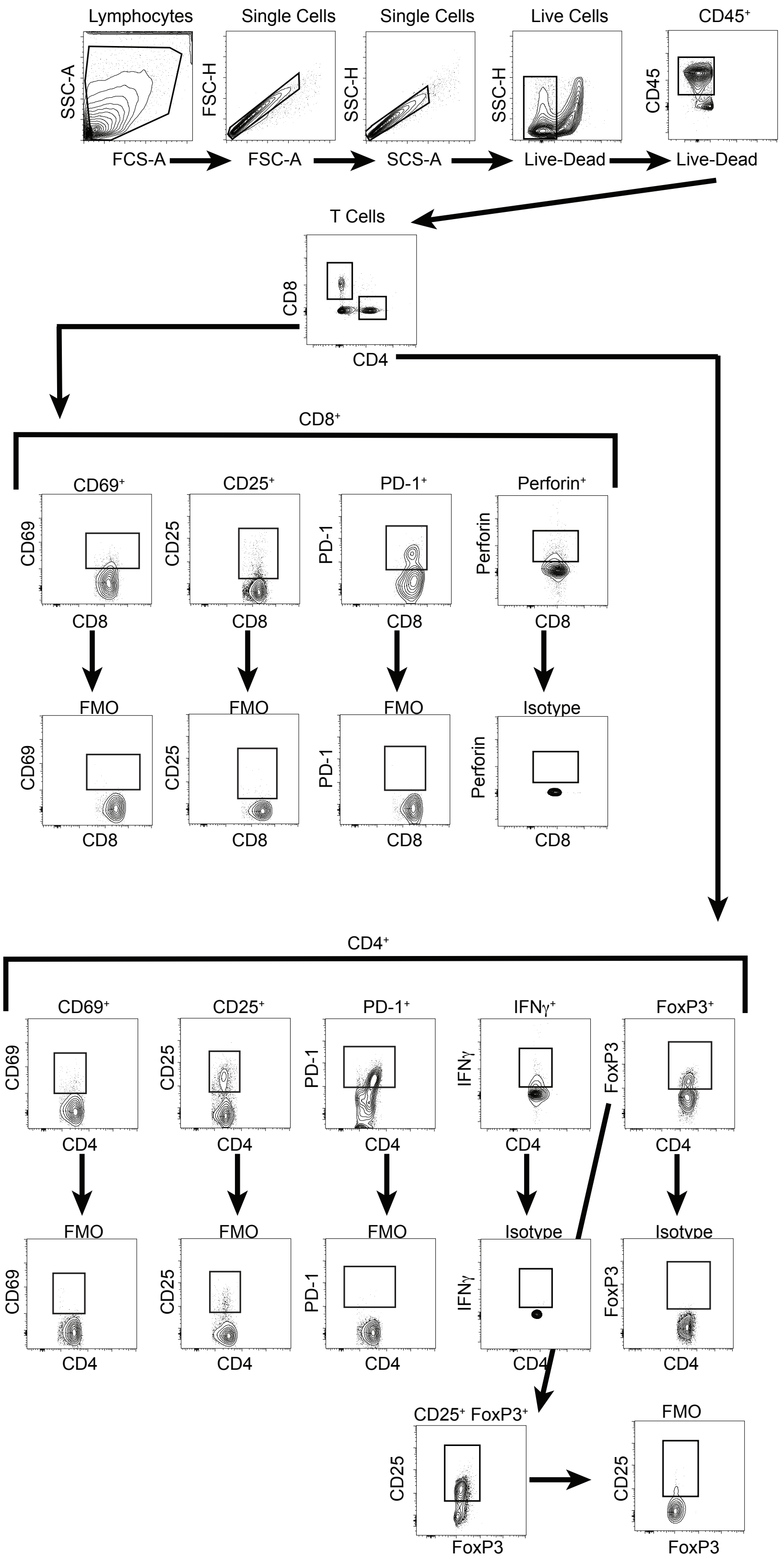

Supplemental Figure 6: Flow cytometry gating strategy of T cells.

Cells were pre-gated on single cells, live cells, and $CD45^+$ cells for immunophenotyping of CD4 T cells (CD4) and CD8 T cells (CD8). Fluorescence minus one (FMO) is also shown for CD69, CD25, PD-1. Isotype is shown for intracellular staining for FoxP3, IFNγ, and perforin.

(A)

Reagent	Clone	Company	Catalog #	Dilutions	RRID
BD Golgi plug	N/A	BD Bioscience	BDB555029	1 µL/mL	AB_2869014
FC Block	N/A	Biocell Technology	BE0307	1 µg/mL	AB_2736987
Live/Dead	N/A	ThermoFisher	L-34976	0.067 µL/test	N/A
CD45	30-F11	Biolegend	103108	0.156 µL/test	AB_312973
F4/80	BM8	Ebioscience	25-4801-82	0.313 µL/test	AB_469653
CD86	PO3.1	Ebioscience	12-0861-81	0.313 µL/test	AB_465764
CD80	16-10A1	BD Bioscience	560526	0.156 µL/test	AB_1727514
MHC-II Ag^{7}	OX-6	BD Bioscience	744130	0.156 µL/test	AB_2742020
LY6C	HK1.4	Biolegend	128036	1 µL/test	AB_2562353
LY6G	1A8	Biolegend	127628	0.625 µL/test	AB_2562567
CD45R/B220	RA3-6B2	BD Bioscience	553092	0.625 µL/test	AB_398531
CD11b	M1-70	BD Bioscience	563015	0.313 µL/test	AB_2737951
CD4	GK1.5	BD Bioscience	563106	0.156 µL/test	AB_2687550
CD8α	53-6.7	BD Bioscience	563898	0.313 µL/test	AB_2738474
CD69	H1.2F3	Ebioscience	15-0691-82	0.156 µL/test	AB_468772
CD25	PC61	BD Bioscience	561780	0.156 µL/test	AB_10893596
PD-1	29F.1A12	Biolegend	135220	0.313 µL/test	AB_2562616
IFNγ	XMG1.2	BD Bioscience	564336	10 µL/test	AB_2738752
Perforin	S16009B	Biolegend	154406	10 µL/test	AB_2721641
FoxP3	FJK-16s	Ebioscience	12-5773-80	10 µL/test	AB_465935
TNF	MP6-XT22	BD Bioscience	560659	4 µL/test	AB_1727580

(B)

**Reagent**	**Clone**	**Company**	**Catalog #**	**Dilutions**	**RRID**
anti-MDA5	D74E4	Cell Signaling	5321S	1:1000	AB_10694490
anti-MDA5	N/A	Fisher Scientific	PIPA5-89344	1:500	AB_2805507
anti-RIG-I	D14G6	Cell Signaling	3743S	1:1000	AB_2269233
anti-pSTAT1 (Y701)	58D6	Cell Signaling	9167	1:1000	AB_561284
anti-STAT1	N/A	Cell Signaling	9172	1:1000	AB_2198300
anti-β-actin	AC-15	Sigma Aldrich	A5441	1:10000	AB_476744
anti-rabbit	N/A	LI-COR	926-68073	1:20000	AB_10954442
anti-rabbit	N/A	LI-COR	926-32213	1:20000	AB_621848
anti-mouse	N/A	LI-COR	926-32212	1:20000	AB_621847

Supplementary Table 1.
Reagents for flow cytometry (A) and Western blot (B)

Full unedited blots for Figures 5 and 7

Full unedited blot for Figure 5A

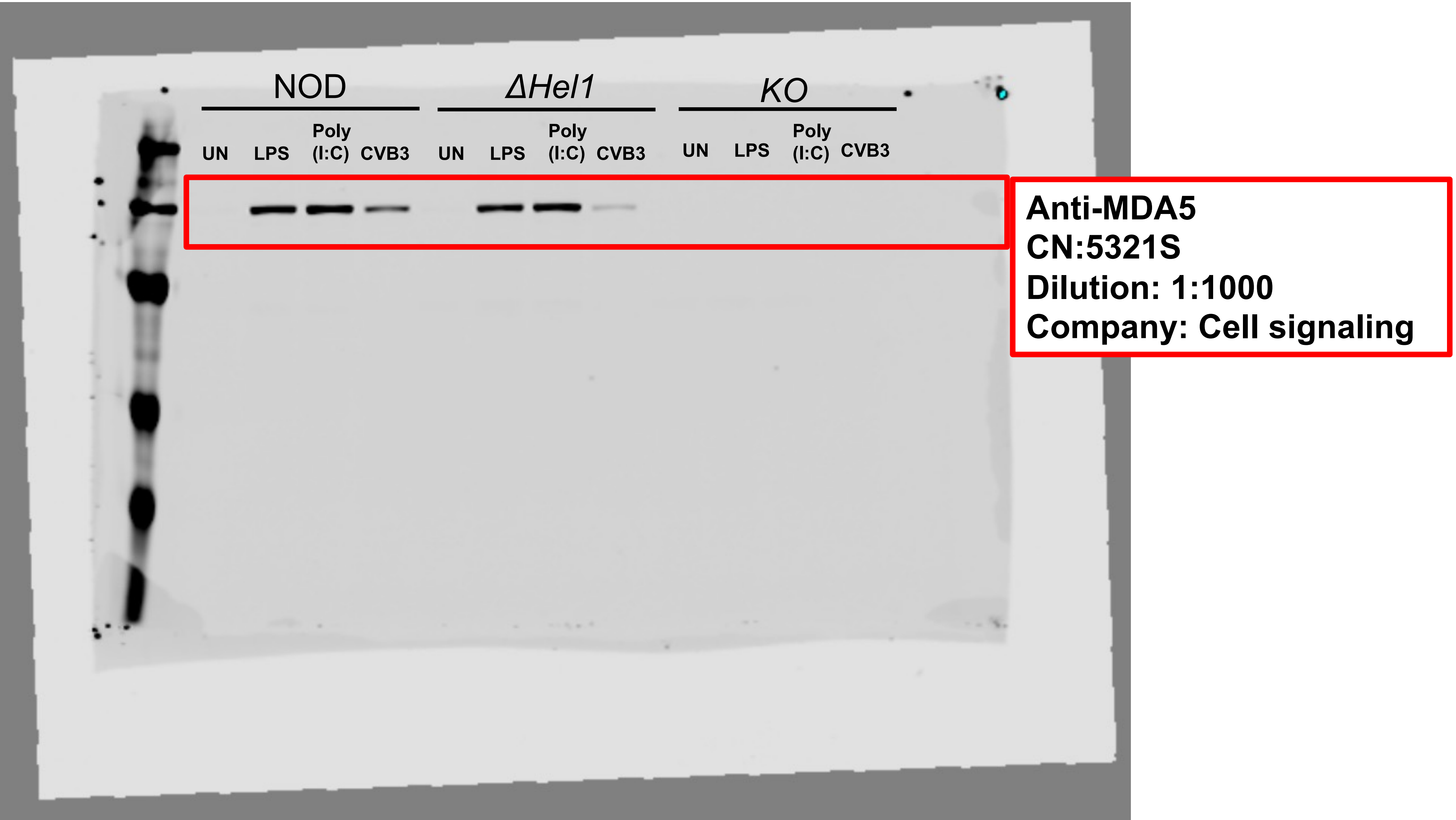

Full unedited blot for Figure 5A

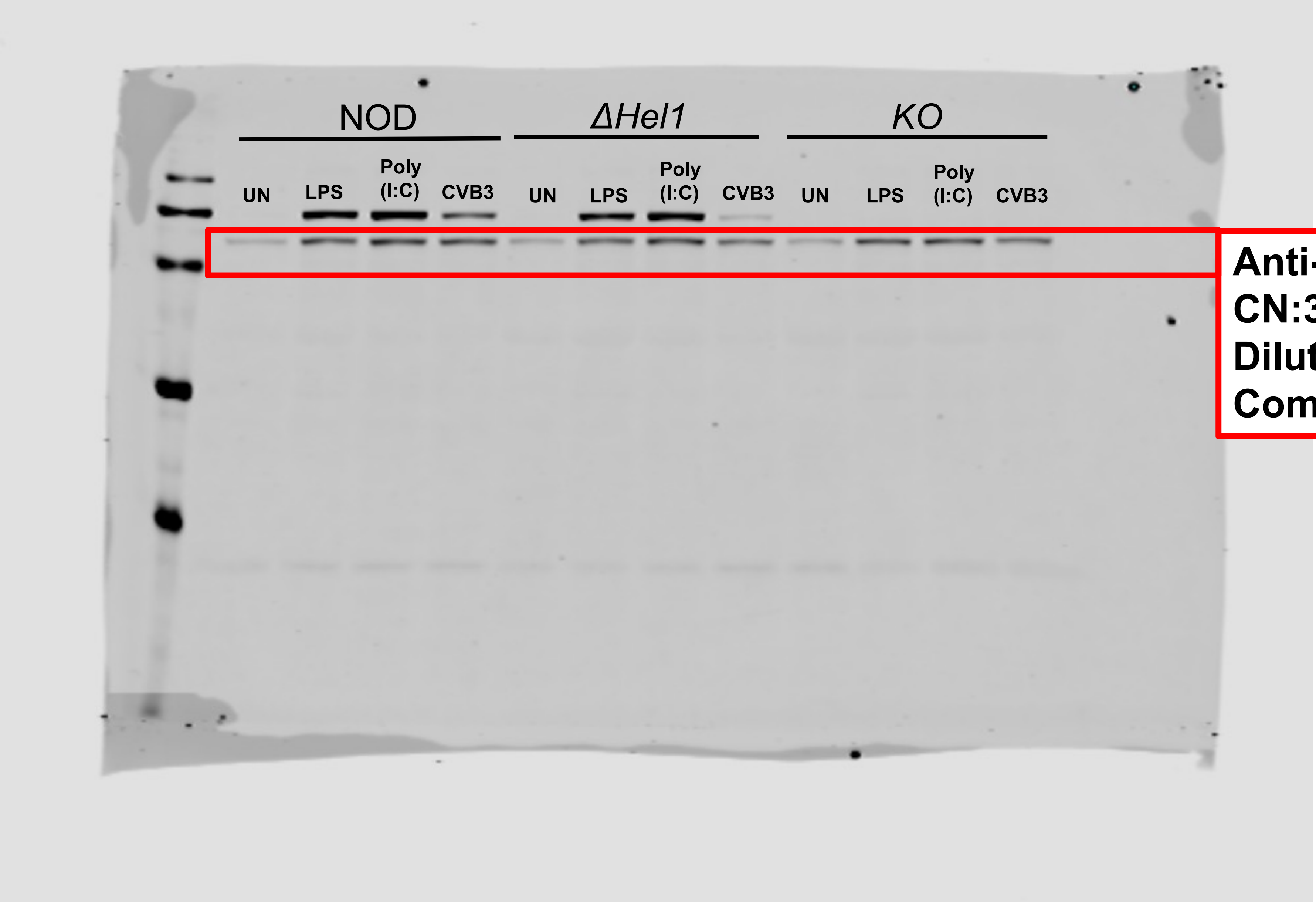

Anti-RIG-I
CN:3743S
Dilution: 1:1000
Company: Cell signaling

Full unedited blot for Figure 5A

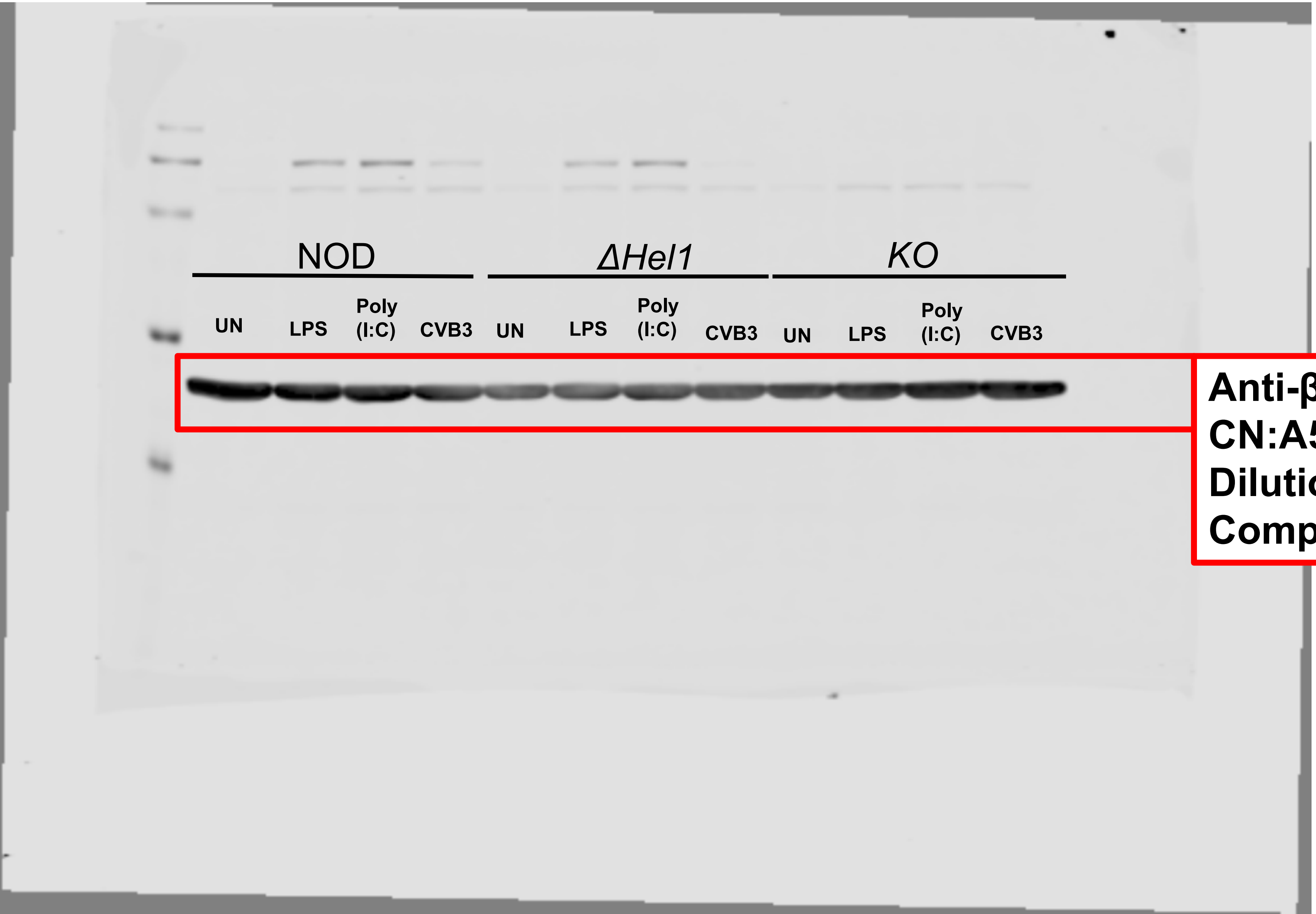

Anti-β-actin
CN:A5441
Dilution: 1:10000
Company: Sigma Aldrich

Full unedited blot for Figure 5A

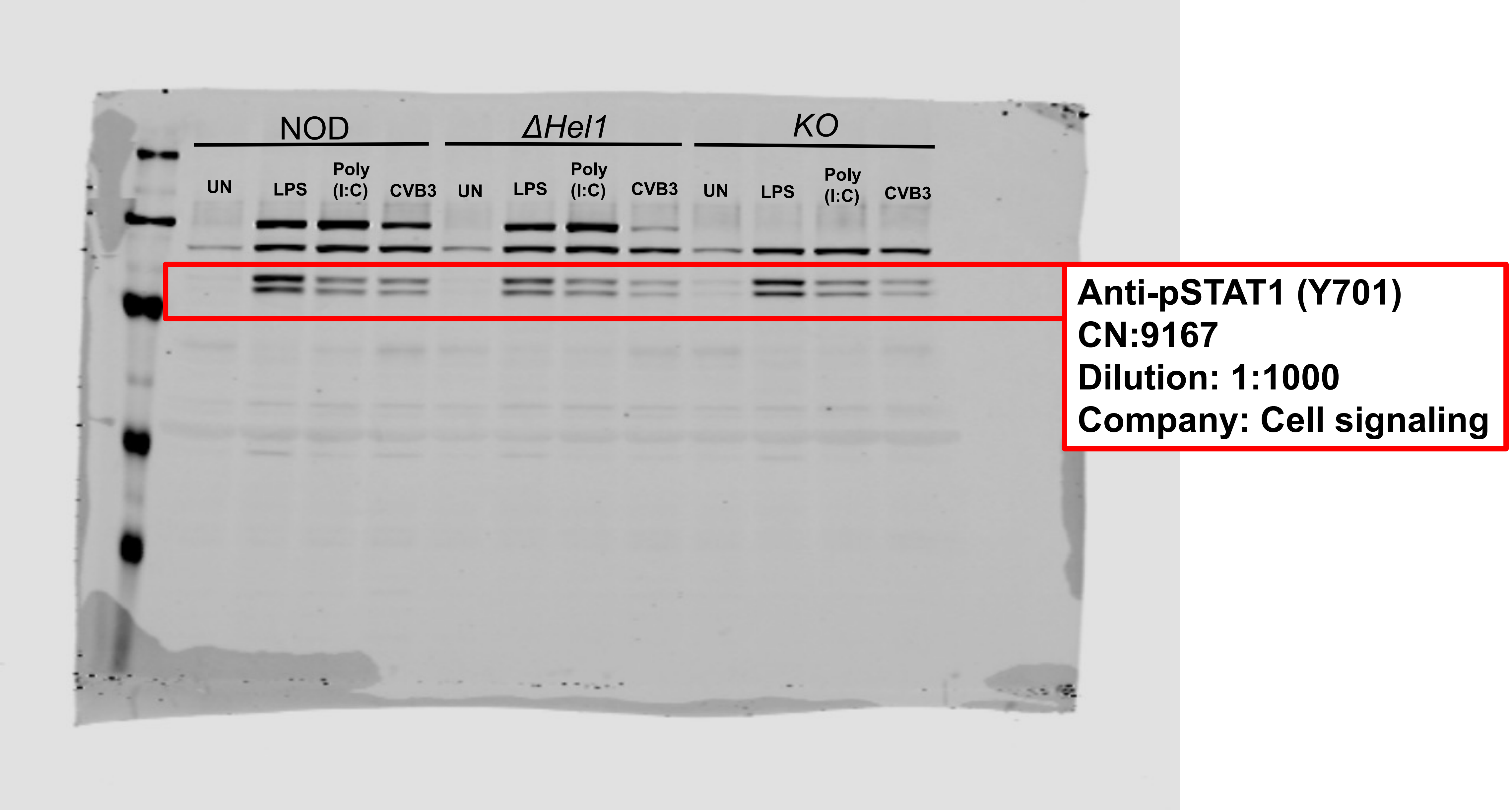

Full unedited blot for Figure 5A

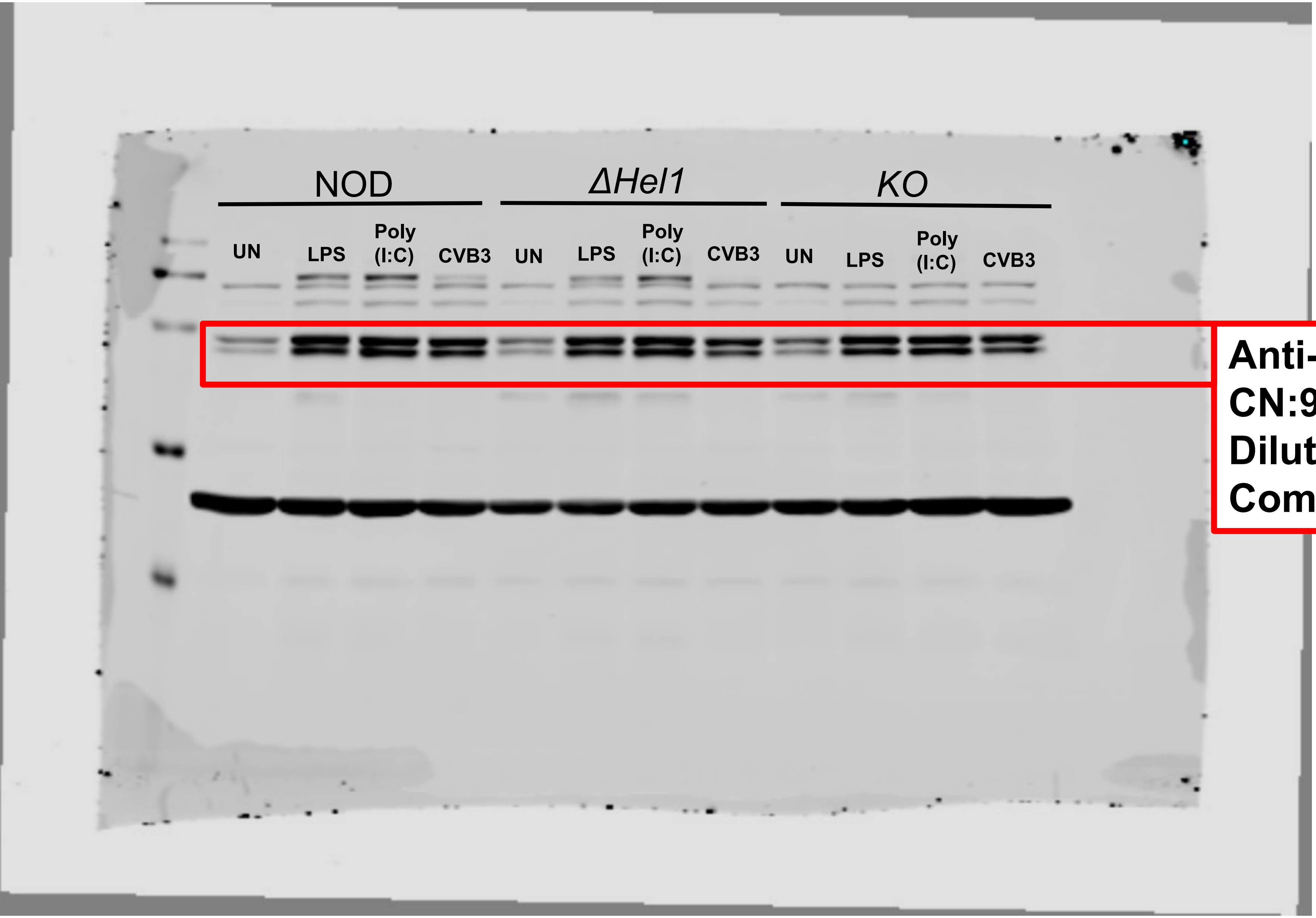

Anti-STAT1
CN:9172
Dilution: 1:1000
Company: Cell signaling

Full unedited gel for Figure 7A

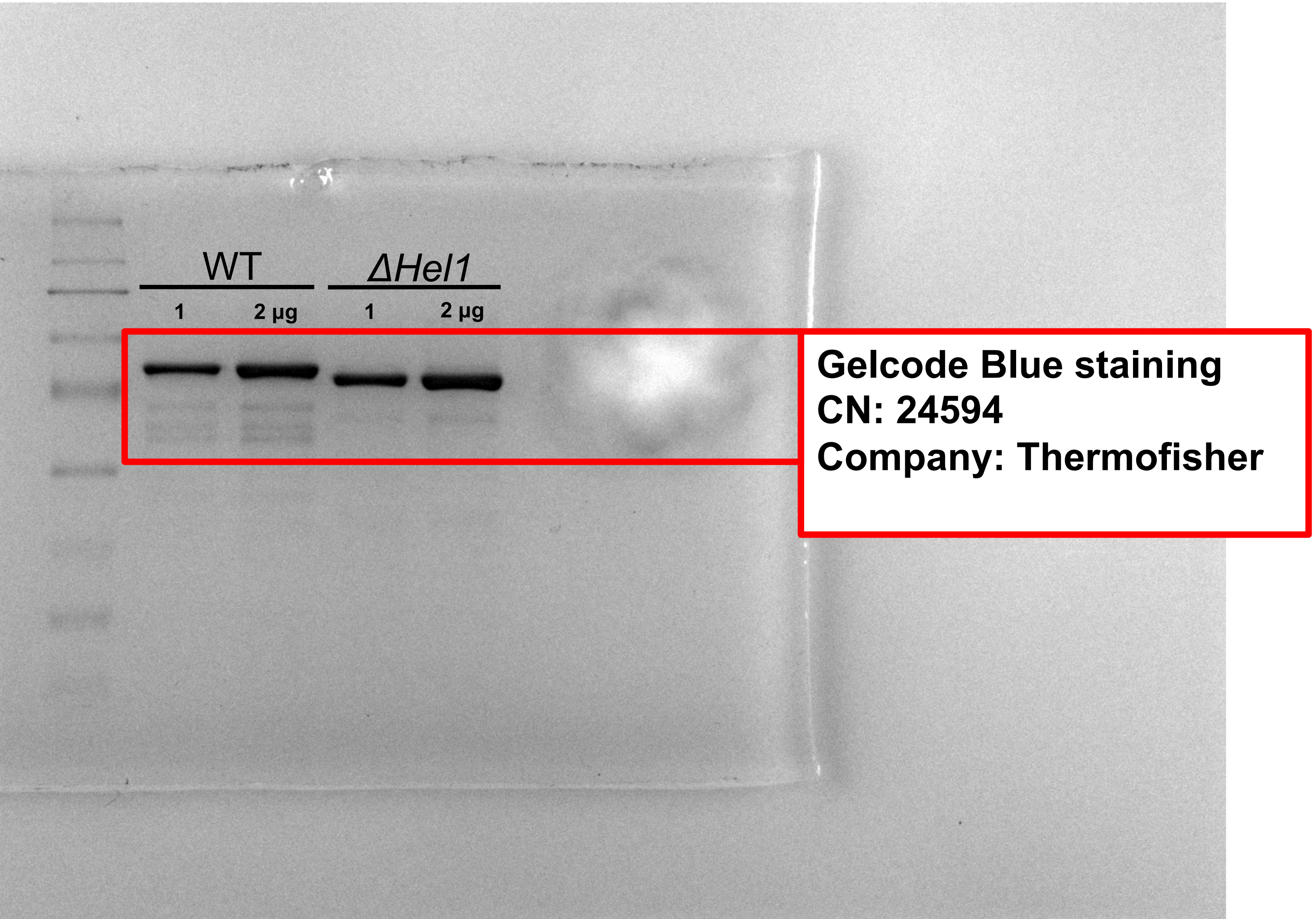

Full unedited blot for Figure 7B

(not overexposed)

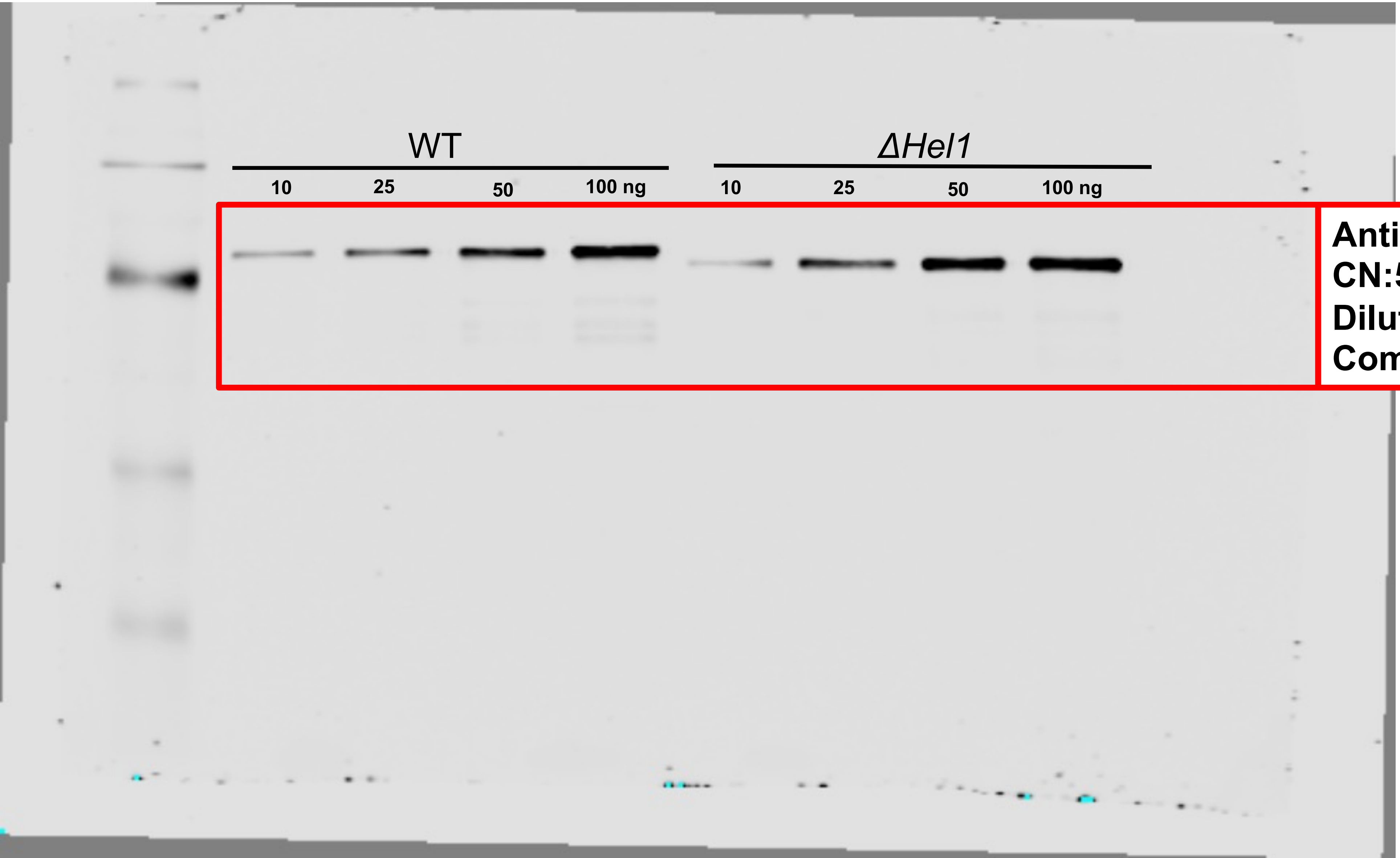

Anti-MDA5
CN:5321S
Dilution: 1:1000
Company: Cell signaling

Full unedited blot for Figure 7B
(Overexposed)

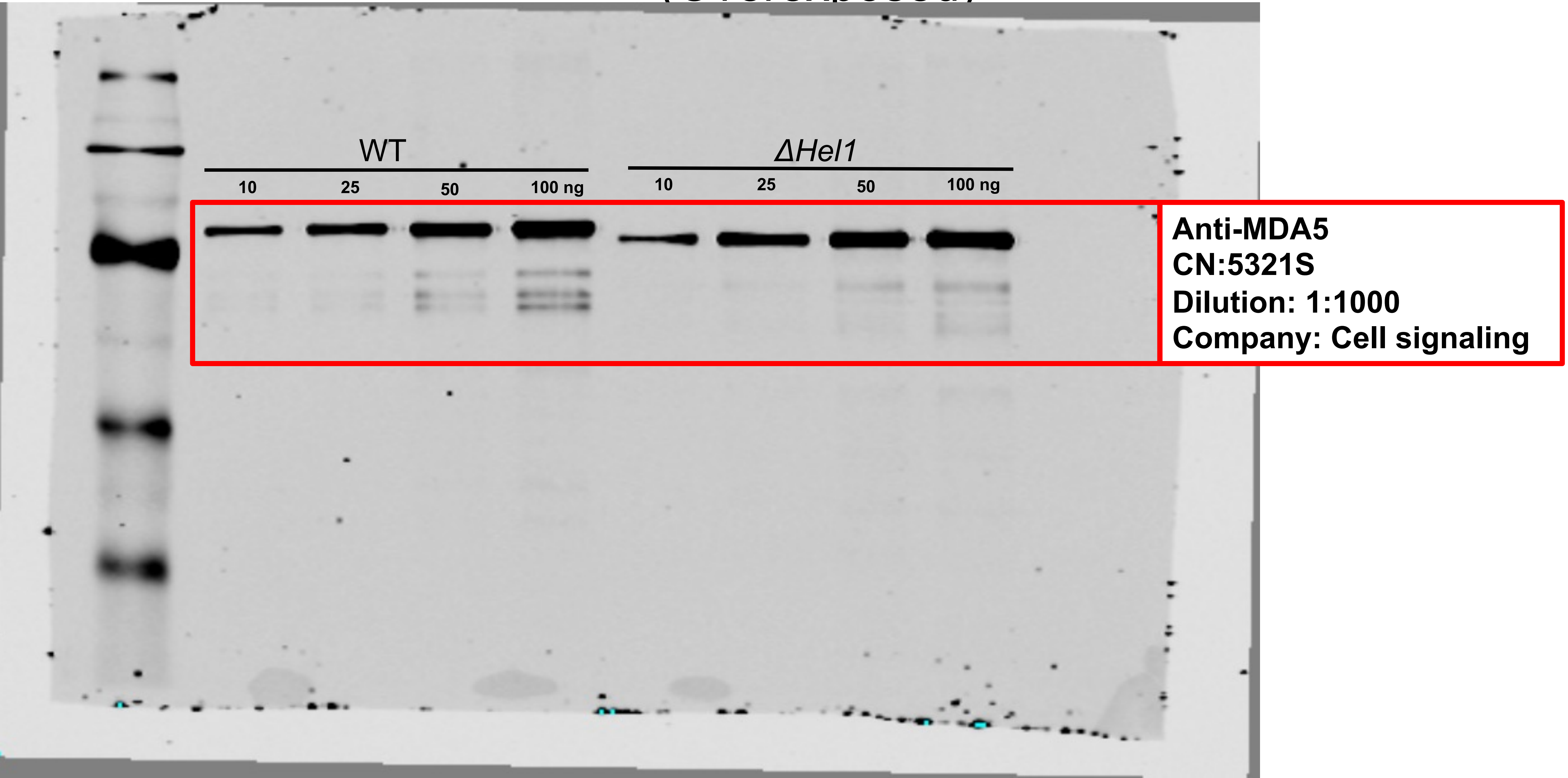

Full unedited gel for Supplemental Figure 4

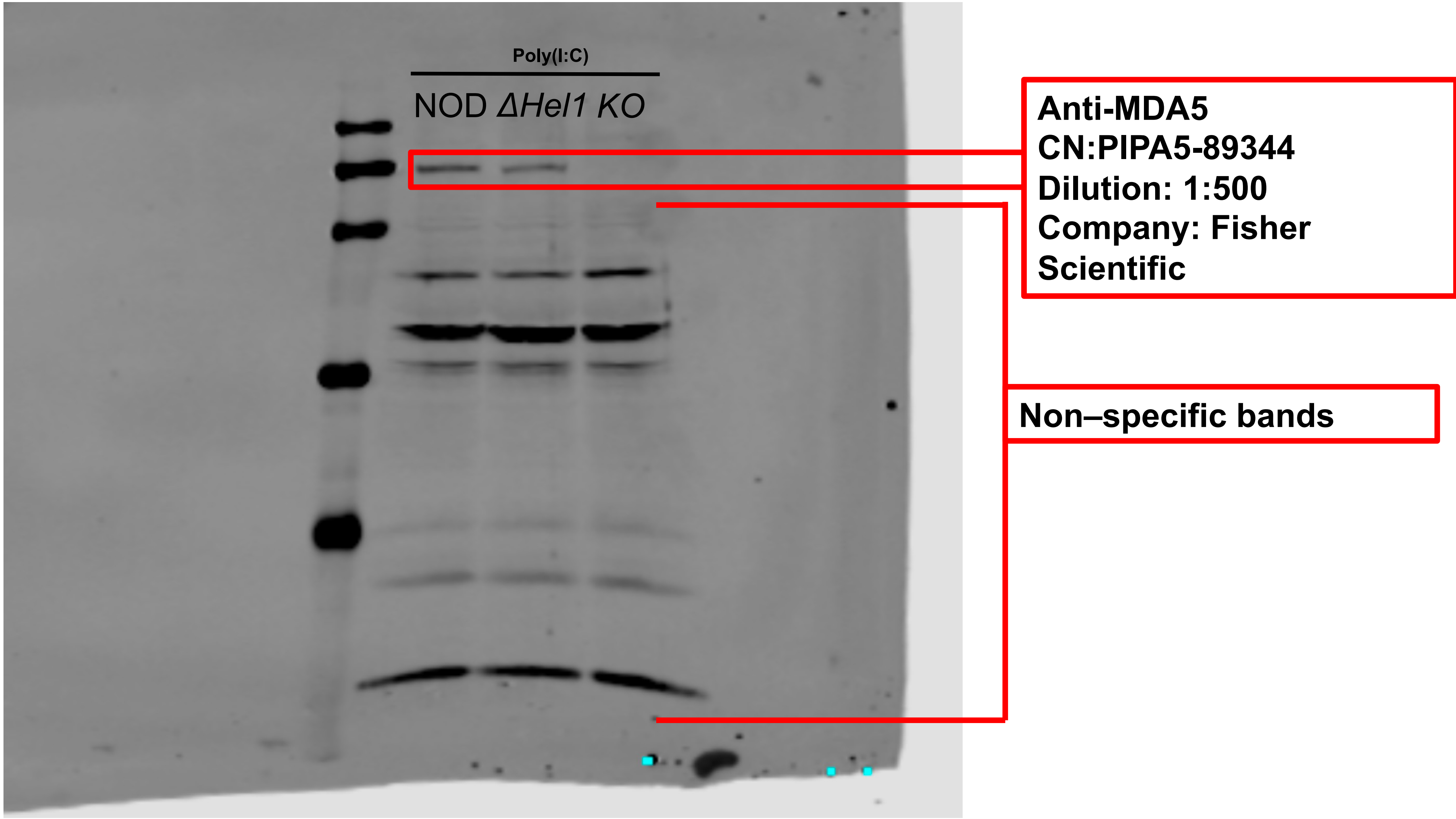

Supplement: Supplemental data [file jciinsight-8-157929-s019.pdf]
